# Metal/oxide interfacial effects on the selective oxidation of primary alcohols

**DOI:** 10.1038/ncomms14039

**Published:** 2017-01-18

**Authors:** Guofeng Zhao, Fan Yang, Zongjia Chen, Qingfei Liu, Yongjun Ji, Yi Zhang, Zhiqiang Niu, Junjie Mao, Xinhe Bao, Peijun Hu, Yadong Li

**Affiliations:** 1 Department of Chemistry, Tsinghua University, Beijing 100084, China; 2 State Key Laboratory of Catalysis, CAS Center for Excellence in Nanoscience, Dalian Institute of Chemical Physics, Chinese Academy of Sciences, Dalian 116023, China; 3 Key Laboratory for Advanced Materials, Center for Computational Chemistry and Research Institute of Industrial Catalysis, East China University of Science and Technology, Shanghai 200237, China; 4 School of Chemistry and Chemical Engineering, The Queen's University of Belfast, Belfast BT9 5AG, UK; 5 University of Chinese Academy of Sciences, Beijing 100049, China; 6 Collaborative Innovation Center for Nanomaterial Science and Engineering, Tsinghua University, Beijing 100084, China

## Abstract

A main obstacle in the rational development of heterogeneous catalysts is the difficulty in identifying active sites. Here we show metal/oxide interfacial sites are highly active for the oxidation of benzyl alcohol and other industrially important primary alcohols on a range of metals and oxides combinations. Scanning tunnelling microscopy together with density functional theory calculations on FeO/Pt(111) reveals that benzyl alcohol enriches preferentially at the oxygen-terminated FeO/Pt(111) interface and undergoes readily O–H and C–H dissociations with the aid of interfacial oxygen, which is also validated in the model study of Cu_2_O/Ag(111). We demonstrate that the interfacial effects are independent of metal or oxide sizes and the way by which the interfaces were constructed. It inspires us to inversely support nano-oxides on micro-metals to make the structure more stable against sintering while the number of active sites is not sacrificed. The catalyst lifetime, by taking the inverse design, is thereby significantly prolonged.

## Abstract (web summary)

Metals supported on metal oxides are common heterogeneous catalysts. Here the authors show that metal/oxide interfacial sites are highly active for alcohol oxidation—independent of the particle sizes—and use this information to design inverse oxide-on-metal particles with high activity and stability.

Aldehydes and ketones are widely used as solvents, polymer precursors, high value-added components in perfumes and intermediates of dyes as well as pharmaceuticals^1^,^2^. Many of them are synthesized by stoichiometric oxidation of alcohols using expensive and toxic oxidants such as chromate or permanganate^1^. Gas-phase selective oxidation of alcohols using oxygen or air as the oxidant represents an industrially feasible and environmentally benign protocol, which produces aldehydes and ketones in a solvent-free, continuous manner^3^,^4^. Although a number of heterogeneous catalysts, such as supported Cu (ref. 3), Ag (ref. 5), Au (ref. 6) and bimetallic particles (ref. 6), have been developed for this process, the nature of the active sites are still not fully understood and many of the catalysts suffered fast degradation in activity.

The pivotal role of metal/oxide interfaces has been recognized in several catalytic processes. For example, Cu/ZnO/Al_2_O_3_ and Cu/CeO_*x*_ are highly active for methanol synthesis^7^; ZnPd/ZnO leads to high CO_2_ selectivity in methanol steam reforming^8^; Cu/CeO_*x*_ (ref. 9), Au/CeO_*x*_ (ref. 10), Pt/CeO_*x*_ (ref. 11) and PtCo_*x*_/Co_3_O_4_ (ref. 12) exhibit improved efficiency for water-gas shift reaction; Pt/FeO_*x*_ (ref. 13), Au/TiO_*x*_ (ref. 14) and Au/FeO_*x*_ (ref. 15) boost CO (preferential) oxidation. In the context of alcohol oxidation, Abad *et al*.^16^,^17^ observed high and general activity of Au/CeO_2_ under solvent-free conditions as well as in organic solvents. Their study established a linear correlation between the activity and the total number of external gold atoms, as well as the surface coverage of the cerium support. They proposed that the cerium support stabilize positive gold species which act as Lewis-acid sites to adsorb alcohols, and also facilitate oxygen activation through oxygen vacancies to promote the re-oxidation of preformed metal hydrides. Bauer *et al*.^18^ developed an efficient Au-CuO_*x*_ core–shell catalyst for aerobic oxidation of ethanol. They presumed that the close contact between the metal core and oxide shell generates the active sites. Further mechanistic explanation was attempted by a recent study by Redina *et al*. They suggested the strong interaction between metallic Au and the isolated Cu^2+^ ions account for the high activity of the Au–Cu catalysts^19^.

Here, we report gas-phase selective oxidation of primary alcohols over a series of metal/oxide interfaces with greatly enhanced performance. The underlying mechanism of the promoted activity at the interfacial sites is investigated by scanning tunnelling microscopy (STM) measurements on both FeO/Pt(111) and Cu_2_O/Ag(111) model catalysts together with theoretical studies. Several common features of different metal/oxide systems are further disclosed after analysing the overall reaction carefully by density functional theory (DFT) calculations. On the basis of these understanding, we conceive an anti-sintering catalyst and extend the concept to a practical monolithic catalytic system based on TMO/Ag/Cu-gauze (transition metal oxide) structure.

## Results

### STM and DFT studies on FeO/Pt(111) model catalyst

**Figure 1:**
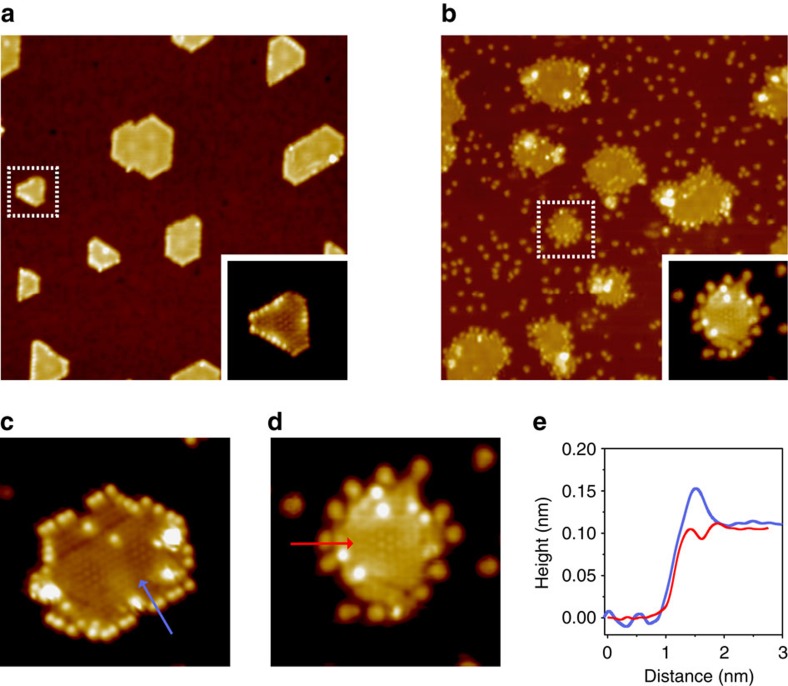
STM images of benzaldehyde formation at the FeO/Pt(111) interface. (**a**) FeO islands on Pt(111) expose O-terminated steps after the annealing in 1.0 × 10^−7^ mbar O_2_ at 400 K. (**b**) FeO nanoislands with O-terminated steps after the exposure of ∼1 L benzyl alcohol at 300 K, followed by flash annealing to 350 K. (**c**) FeO nanoisland with Fe-terminated edge after the exposure of ∼1 L benzyl alcohol at 300 K. (**d**) The magnification of an FeO island in **b** with adsorbed species at the FeO/Pt(111) interface. (**e**) Line profiles of adsorbed species: blue line for the species marked in **c** and the red line for the species in **d**. Image sizes and scanning conditions: (**a**) 43 nm × 43 nm, *V*_s_=0.4 V, *I*_t_=0.1 nA; (**b**) 43 nm × 43 nm, *V*_s_=0.6 V, *I*_t_=0.1 nA; (**c**) 8.5 nm × 8.5 nm, *V*_s_=0.1 V, *I*_t_=2.1 nA; (**d**) 7 nm × 7 nm, *V*_s_=0.1 V, *I*_t_=0.7 nA.

We started our investigation on the gas-phase oxidation of benzyl alcohol over Pt, FeO and Pt/FeO colloidal nanoparticles. Individual Pt or FeO shows much lower conversion (<15%) than Pt/FeO nanocomposite (∼86%) at a temperature from 230 to 270 °C (Supplementary Fig. 1A–C). The apparent activation energy of benzyl alcohol oxidation decreases from 91 kJ mol^−1^ on FeO and 102 kJ mol^−1^ on Pt to 64 kJ mol^−1^ on Pt/FeO nanocomposite (Supplementary Fig. 1D). Encouraged by this interesting observation, we constructed FeO islands on the Pt(111) surface, termed as FeO/Pt(111), to study the benzaldehyde formation using STM. The atomic structure of as-prepared FeO islands is magnified in Supplementary Fig. 2. The curvy shape of step edges of FeO islands with periodic indentation is a typical character for the Fe-terminated step edges of FeO on Pt(111)^20^ (denoted as Fe-FeO/Pt(111)). When exposed to O_2_, the Fe-terminated step edges evolve into O-terminated step edges (denoted as O-FeO/Pt(111), Fig. 1a) by O_2_ dissociation at the coordinatively-unsaturated Fe sites. These steps were found to be the most stable step termination when FeO/Pt(111) was exposed to O_2_ at room temperature or above^21^. The evolution of the oxidation state of iron was investigated by X-ray photoelectron spectra (XPS). Upon oxygen adsorption, two-coordinated ferrous sites at the steps of FeO become three-coordinated, as is the case for Fe atoms inside the FeO nanostructures. Thus, FeO should remain the Fe^2+^ state with oxygen attached to the step edges. Accordingly, in the XPS spectra (Supplementary Fig. 3), Fe 2p_3/2_ peak from O-FeO/Pt(111) (709.3 eV) is only slightly more positive than that of Fe-FeO/Pt(111) (708.6 eV), indicating the Fe^2+^ state of FeO nanostructures, regardless of their step termination. Fe^3+^ on Pt(111) should give a Fe 2p_3/2_ peak at above 710 eV, as shown in the previous study^22^. Supplementary Figure 4 shows the surface of O-FeO/Pt(111) after an exposure of ∼0.4 L (1 L=1.0 × 10^-6^  Torr s) benzyl alcohol at room temperature. The adsorbed species appear to be bright spots on both the Pt(111) surface and the edges of O-FeO islands. Notably, the density of bright spots appears to be more enriched at the step edges of O-FeO islands with smaller size, as marked by circles in Supplementary Fig. 4A. The density of bright spots increases with the increasing exposure of benzyl alcohol (1 L, Fig. 1b). From image analysis, the spot density at the interface corresponds to 0.132 molecule per O/Fe site, while on the Pt(111) surface the number is 0.0096 molecule per Pt site (see detailed analyses in Supplementary Fig. 5). The interfacial adsorption of benzyl alcohol is further compared at Fe-terminated steps (Fig. 1c) and O-terminated steps (Fig. 1d). Benzyl alcohol could stably adsorb at O-FeO steps after the annealing at 350 K (Fig. 1b), whereas at Fe-FeO steps, benzyl alcohol could adsorb at 300 K, but desorb at 350 K (Supplementary Fig. 6). In STM images, benzyl alcohol appears to have a higher apparent height at Fe-FeO steps than at O-FeO steps (Fig. 1e). The species with lower apparent height at the O-FeO steps could be assigned as dissociatively adsorbed benzyl alcohol species, which could be distinguished from molecularly adsorbed benzyl alcohol because of their different apparent heights and desorption temperatures. Due to the dissociation of O–H bond, oxygen tends to withdraw electrons from neighboring atoms, rendering a lowered apparent height of the adsorbate in STM topography. Such character has also been used to distinguish molecular or dissociated alcohol species previously^23^. The facile deprotonation of alcohols on FeO/Pt(111) has been shown in IRAS and TPD studies by Kim *et al*.^24^ A broad water desorption peak at between 200 and 400 K, indicating the facile deprotonation of alcohols, was observed, which does not occur on Pt(111). Accordingly, our STM images suggest that H from benzyl alcohol reacts with O at the edge of FeO to form water, which desorbs at above 200 K. Indeed, under-coordinated oxygen at the edge of FeO is very reactive that, upon the flash to 350 K, part of the edges of FeO islands are reduced (Fig. 1).

**Figure 2:**
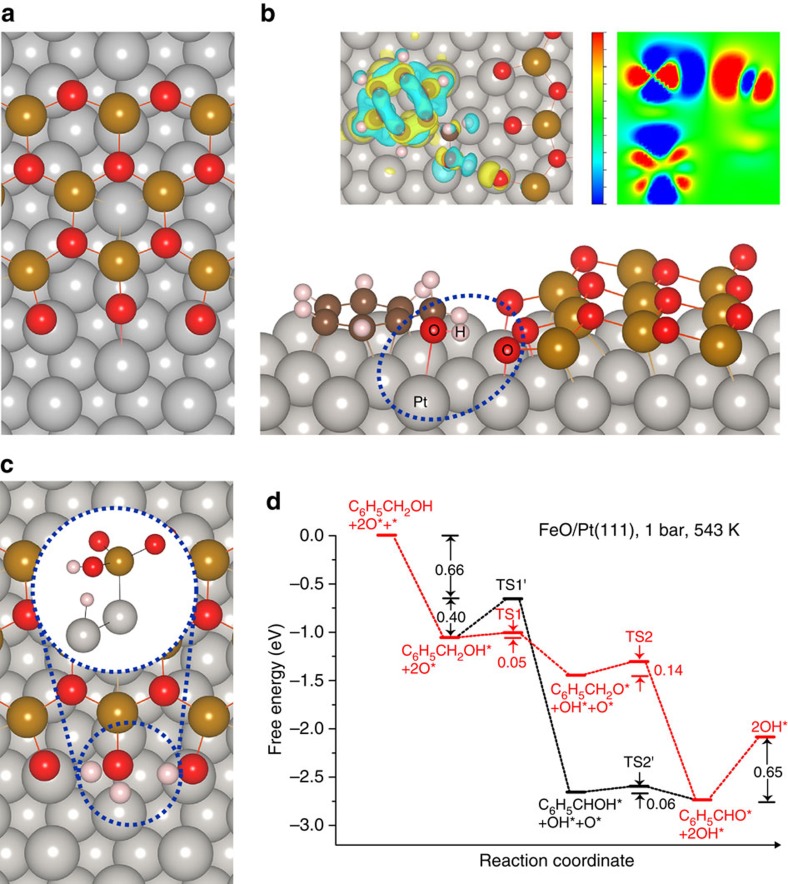
Adsorption configurations and free energy profile of benzaldehyde formation at the FeO/Pt(111) interface. (**a**) Oxygen-terminated FeO/Pt(111) interface (O-FeO/Pt(111)). (**b**) Benzyl alcohol adsorption at the O-FeO/Pt(111) interface. The inserts depict the 3D and 2D isosurfaces of charge density difference for benzyl alcohol adsorption, respectively, in which yellow and red indicates the electronic accumulation and blue for electronic depletion. The relevant atoms in 2D isosurface represent for the interaction of hydroxyl with boundary oxygen and substrate Pt (in blue ellipse). (**c**) Transition state structure of water formation (O–H distance is 1.58 Å). The inset depicts the side view of reaction centre. (**d**) Free energy profile of benzaldehyde formation at FeO/Pt(111) interface. The red and black lines represent for pathway 1 and 2, respectively. In **a**–**c**, the gray balls represent for Pt atoms, red for O, light-pink for H, dark-goldenrod for Fe and sienna for C.

To provide insight into the mechanism of benzaldehyde formation, first-principles calculations were carried out on both the Pt(111) surface and the FeO/Pt(111) interface. The free energy profiles of benzaldehyde formation on Pt(111) are illustrated in Supplementary Fig. 7 and discussed in detail in the Supplementary Discussion. The Fe-terminated FeO/Pt(111) interface was constructed by placing a thin FeO ribbon on Pt(111) (Supplementary Fig. 8)^21^. In our model, the FeO/Pt ribbon can maintain its hexagonal layer configuration during the optimization calculation, indicating that the FeO layer is reasonably stable. The exposed coordinatively-unsaturated Fe sites show a strong oxygen adsorption ability (−1.27 eV) and could readily be oxidized via a low oxygen dissociation barrier (0.34 eV, Supplementary Fig. 9), resulting in the O-FeO/Pt(111) interface (Fig. 2a). This is in accordance with our STM observation and previous results^21^. On the O-FeO/Pt(111) interface, the hydrogen of hydroxyl in adsorbed benzyl alcohol forms a hydrogen bond with a terminated oxygen (Fig. 2b; Supplementary Fig. 10A). The adsorption of benzyl alcohol is exothermic by 3.62 eV, slightly stronger than that on the Fe-FeO/Pt(111) interface (Supplementary Fig. 11, 3.38 eV). Interestingly, with the aid of the interface oxygen, the O-H bond activation barriers are significantly diminished to below 0.1 eV for both reaction pathways shown in Supplementary Table 1. In pathway 1 (Supplementary Fig. 10B–D), the subsequent C–H bond cleavage can also occur facilely via a low reaction barrier (0.14 eV) with 1.29 eV heat releasing; while in pathway 2 (Supplementary Fig. 10E–H), the activation of C–H bond cleavage is much more difficult (0.40 eV). Clearly, pathway 1 is favoured for the benzaldehyde formation due to a lower overall activation barrier. After the O–H and C–H dissociations, the O-FeO/Pt is converted to HO-FeO/Pt (Fig. 2c) and surface H atoms are produced. Water molecules are then formed through hydrogenating OH by the surface H atoms on the interface to restore the Fe-terminated FeO/Pt interface (Supplementary Fig. 12), completing the catalytic cycle. It is found that the overall activation energy of water formation is below 0.4 eV (Supplementary Table 2), which is not difficult to overcome under the experimental conditions.

By comparing the Pt(111) surface to the FeO/Pt interface, the following striking features can be seen: firstly, the benzyl alcohol adsorption is much stronger on the FeO/Pt interface, indicating that the benzyl alcohol coverage would be increased at the FeO/Pt interface, which coincides well with the STM results. Secondly, for the slow step of O–H bond breaking, the reaction barrier is significantly lower at the O-FeO/Pt(111) interface than on Pt(111) (0.05 eV versus 0.70 eV, Supplementary Fig. 13 and Supplementary Table 3). This is also evidenced by our STM study of the apparent heights of adsorbed species at the O-FeO/Pt(111) interface. It is clear that the strong hydrogen binding between the oxygen at the edge of FeO/Pt interface and the hydrogen of hydroxyl not only increases the adsorption of benzyl alcohol but also effectively weakens the O–H bond of benzyl alcohol, which results in a decrease of the activation barrier. Similar to the O–H bond breaking, the barrier of C–H scission on the interface is also considerably reduced comparing to Pt(111) (0.14 eV versus 0.42 eV), leading to the high overall activity.

### Quantitative study on *in situ* formed PdCu_*x*_/Cu_2_O catalyst

**Figure 3:**
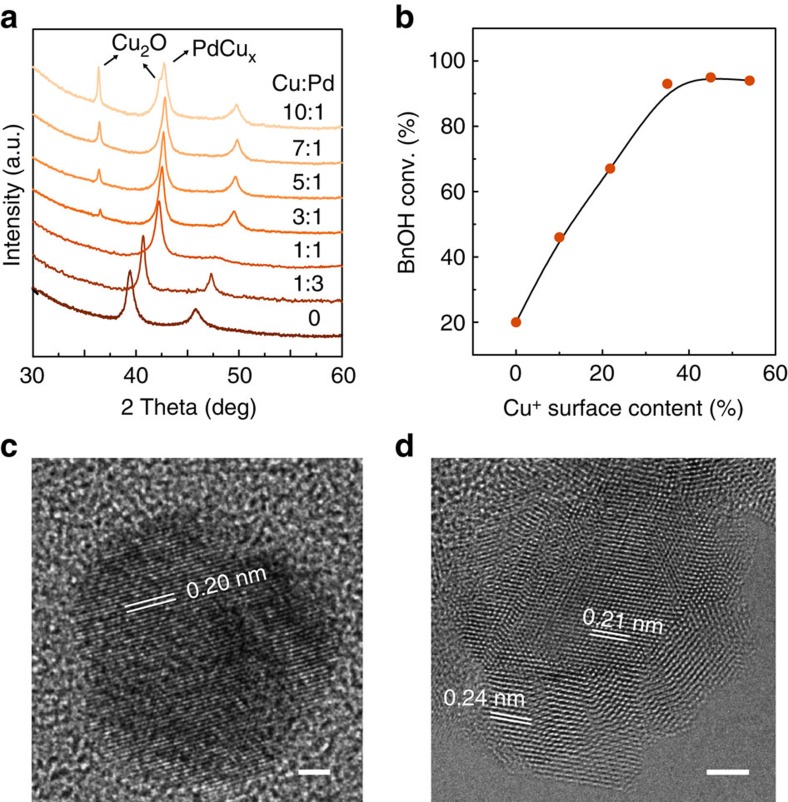
**Dependence of catalytic activity on the surface Cu** ^**+**^ **species of PdCu**_***x***_ **–Cu**_**2**_ **O nanocomposites evolved from fresh Pd-Cu** **alloy.** (**a**) X-ray diffraction patterns of the used Pd-Cu alloy catalysts. (**b**) The relationship between surface Cu^+^ content and benzyl alcohol conversion. (**c**) HRTEM image of the fresh PdCu_7_ catalyst. (**d**) HRTEM image of the used PdCu_7_ catalyst exhibiting the phase segregation of PdCu_*x*_ and Cu_2_O. Scale bar, 2 nm.

The interface-enhanced benzyl alcohol oxidation was quantitatively studied on PdCu_*x*_/Cu_2_O interface which evolved from Pd-Cu alloy under reaction conditions. A series of monodisperse Pd-Cu alloys with tunable compositions (Supplementary Figs 14 and 15) were prepared by colloidal synthesis. Using pure Pd as a catalyst, benzyl alcohol conversion is 20% while the selectivity of undesired benzene is up to 9% (Supplementary Table 4, entry 1). However, after incorporating Cu into Pd nanoparticles, the catalyst performance is progressively enhanced (entries 2–7) with the increase of Cu:Pd molar ratio. Notably, with the Cu:Pd ratio above 5:1, the benzyl alcohol conversion and the benzaldehyde selectivity are increased to >93 and >97%, respectively (entries 5–7). Nevertheless, the benzyl alcohol conversion is as low as 21% when pure Cu was used (Supplementary Fig. 16, and entry 8). X-ray diffraction patterns of the used Pd-Cu alloy catalysts indicate that the fresh Pd-Cu alloy (Supplementary Fig. 17) is transformed into PdCu_*x*_/Cu_2_O heterostructures (Fig. 3a), and the intensity of Cu_2_O phase increases along with the Cu:Pd molar ratio. XPS also shows the presence and monotonic increase of Cu^+^ over the used catalyst surface (Supplementary Figs 14 and 18). More importantly, we found that the benzyl alcohol conversion increased linearly at low surface Cu^+^ content, and then maintained with a further increase of surface Cu^+^ content (Fig. 3b). High-resolution transmission electron microscopic (HRTEM) images of PdCu_7_ catalysts before and after the catalytic reaction visualize the structural evolution from homogeneous alloy to the heterostructure of small Cu_2_O patches on PdCu_*x*_ particles (Fig. 3c,d; Supplementary Fig. 19). Additional control experiments were further performed, as shown in Supplementary Table 4. Pure Cu_2_O only delivers a benzyl alcohol conversion of 19% (entry 9). However, the benzyl alcohol conversion is enhanced to >90% (entries 10–13) by even physically mixing Cu_2_O with low active Pd, Pd_3_Cu, PdCu or PdCu_3_. These observations strongly support that the PdCu_*x*_/Cu_2_O interface is the active sites for benzyl alcohol oxidation.

### Validation of interfacial effects on Cu_2_O/Ag(111) catalyst

The metal/oxide interfacial effects on benzyl alcohol oxidation were also validated in inexpensive Ag/TMO system by STM and theoretical studies. We first investigated the benzyl alcohol adsorption at the interface of Cu_2_O/Ag(111) using STM. Supplementary Fig. 20A shows Cu_2_O islands of monolayer thickness dispersed on the Ag(111) substrate. The ordered honeycomb structure of Cu_2_O islands is depicted in Supplementary Fig. 20B, with all Cu atoms resolved as bright dots. The lattice spacing of Cu_2_O on Ag(111) is measured of 0.60 nm, same as that of Cu_2_O(111) on Cu(111)^25^. Supplementary Fig. 20C shows the typical topography of Cu_2_O/Ag(111) after the exposure of benzyl alcohol at 115 K. Benzyl alcohol molecules, appearing as bright spots, adsorb only at edges of Cu_2_O islands (Supplementary Fig. 20D). After the annealing at 300 K, adsorbed benzyl alcohol molecules desorb partially, accompanying the formation of new species at the Cu_2_O–Ag interface (Supplementary Fig. 20E and F). These new species exhibit a smaller apparent size than that of adsorbed benzyl alcohol, and could be attributed as phenol oxy intermediates (Supplementary Fig. 21). We further tested the adsorption and activation of benzyl alcohol at CuO_*x*_/Ag(111) interfaces (Supplementary Figs 22 and 23) by DFT calculations. The adsorption of benzyl alcohol is enhanced by 0.8 eV at CuO_*x*_/Ag interface in comparison with Ag(111) (−1.06 eV); and the barrier of O-H scission is largely diminished (below 0.1 eV), comparing the much higher barrier on Ag(111) (1.56 eV). The STM and DFT results demonstrate a similar role of Cu_2_O–Ag interface as FeO–Pt interface in enhancing the adsorption and activation of benzyl alcohol molecules^26^.

### A common descriptor for catalytic activity

To further understand the promotion effect of the oxide/metal systems, the overall reactions were carefully analysed and some common features were obtained using DFT calculations. Firstly, the activation energy of O–H bond cleavage can be significantly decreased in the presence of boundary oxygen, while much higher barriers need to be overcome on metal substrates, including Pt, Pd and Ag surfaces (Supplementary Table 5). Secondly, the activation energies of C–H bond cleavage are in the range from 0.4 to 0.6 eV at the metal surfaces (Ag(111), Pd(111), Pt(111), see Supplementary Table 5), which should be readily overcome under the reaction conditions (over 500 K). Thirdly and perhaps more importantly, the suitable binding strength of oxygen at interfacial sites is found to be of significance to complete the overall catalytic cycle. On one hand, too weak binding strength of oxygen will make the interfacial sites inert for oxygen adsorption and dissociation. On the other hand, the oxygen vacancy formation (that is, hydroxyl removal in our work) will be difficult if the oxygen binding is too strong. Thus, it is crucial to balance the oxygen adsorption and its further removal at the interfacial sites. In the FeO/Pt system, the free energy changes of oxygen adsorption (1/2 O_2_+*→O*) and its vacancy formation (OH*+H* →H_2_O+2*) were calculated to be −0.47 and −0.76 eV, respectively, which are both favourable thermodynamically. The thermodynamics of the above two elementary steps were also calculated on other oxide/metal systems, as shown in Supplementary Fig. 24. Indeed, the oxygen binding strength and its removal have the similar features: the free energy changes of both reactions are all below zero, suggesting that the oxygen species are active for benzyl alcohol oxidation at the considered MO_*x*_/metal (M=Mn, Fe, Co, Ni, Cu; metal=Pt, Pd, Ag) interfaces. In addition, we also considered the systems of MgO/metal. The binding of oxygen is much stronger at the MgO/metal interfacial sites than other MO_*x*_/metal interfaces (Supplementary Fig. 24A). However, the removal of hydroxyl is hindered thermodynamically (Supplementary Fig. 24B), indicating that the overall catalytic cycle will be blocked at MgO/metal interfaces. Experimental results are in good agreement with theoretical prediction. As presented in Supplementary Table 6, the conversion of benzyl alcohol is as low as 12.7% even increasing the reaction temperature to 300 °C. These results suggest that a proper oxygen binding strength is crucial and may be considered as an energy descriptor for such systems.

### The size independence of the interfacial effects

**Figure 4:**
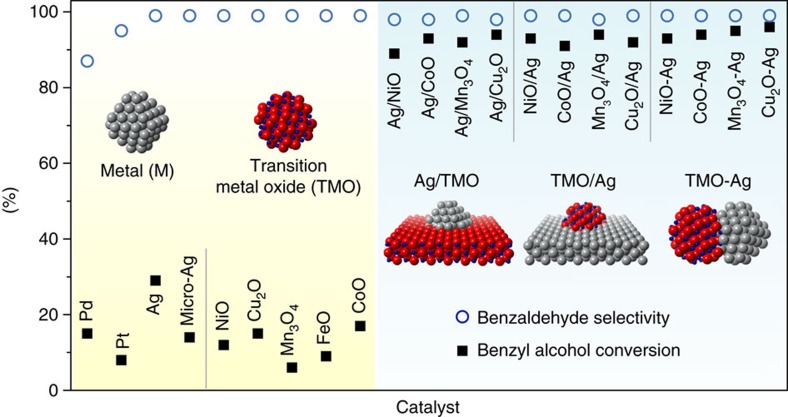
Interfacial effects of three different Ag-TMO structures. Conversion of benzyl alcohol (solid square) and selectivity to benzaldehyde (hollow circle) over metal nanoparticles, TMO nanoparticles, nano-Ag/micro-TMO, nano-TMO/micro-Ag and Ag-TMO nanocomposites (Supplementary Table 7). The results demonstrate that the interfacial effects are independent of component sizes and how they are constructed.

Since the metal–oxide interface has been identified as the active sites, we reason that the aerobic alcohol oxidation on the catalysts would be size insensitive. One would expect similarly high catalytic activity among the catalysts with different dimensions, on condition that sufficient interfacial sites have been constructed. To verify this inference, we designed three different forms of Ag/TMO interfaces including Ag–TMO nanocomposite, nano-Ag supported on micro-TMO, and nano-TMO supported on micro-Ag to demonstrate their equivalency (Supplementary Fig. 25). As shown in Fig. 4 and Supplementary Table 7, Ag–Cu_2_O nanocomposite (Supplementary Fig. 26) exhibits much better performance (Supplementary Table 7, entries 1–3) than pure nano-Ag (Supplementary Fig. 27) and nano-Cu_2_O (Supplementary Fig. 28). The apparent activation energies of benzyl alcohol oxidation decrease from 68 kJ mol^−1^ on nano-Ag and 87 kJ mol^−1^ on nano-Cu_2_O to 43 kJ mol^−1^ on Ag–Cu_2_O nanocomposite (Supplementary Table 8). Even over the physical mixtures of nano-Ag and other nano-TMO such as NiO, CoO, and Mn_3_O_4_ (Supplementary Fig. 25), benzyl alcohol conversions are still much higher than that over the pure nano-Ag or nano-TMO (Supplementary Table 7, entries 4–9). Similar to the nanocomposite, both nano-Ag/micro-TMO (Cu_2_O, NiO, Mn_3_O_4_ and CoO) and inverse nano-TMO/micro-Ag deliver higher benzyl alcohol conversion than individual metal or transition metal oxides (>90% versus <15%, Supplementary Table 7, entries 2 and 10–22). This suggests that the interfacial effects are independent of the size of each constituent. Furthermore, we compared quantitatively the activity of normal nano-metal/micro-oxide and inverse nano-oxide/micro-metal interface by taking the nano-Ag/micro-Mn_3_O_4_ and the inverse nano-Mn_3_O_4_/micro-Ag as a model. We investigated the catalytic performance of the two catalysts with different loadings of nano-Ag and nano-Mn_3_O_4_, respectively. The specific surface areas of as-prepared nano-Ag and nano-Mn_3_O_4_ were determined as 22 and 37 m^2^ g^−1^, respectively, by N_2_ physical adsorption method (Supplementary Table 9). As shown in Supplementary Fig. 29, the benzyl alcohol conversion increases linearly along with the loadings of nano-Ag or nano-Mn_3_O_4_ below 3 wt%, and the slopes are 26 and 42, respectively. The slope ratio (26/42) is almost equal to the specific surface area ratio (22/37), indicating the nearly equivalent catalytic activities at the Ag-Mn_3_O_4_ interface over the two catalysts.

One interesting observation in the Ag/TMO systems is that the physical mixtures of the nano-Ag and nano-TMO, such as Mn_3_O_4_, NiO and CoO, still exhibit enhanced activity over their parent components (Supplementary Table 7, entries 4–6). It probably arises from the *in situ* formed Ag-TMO interface via the close contact of each component. We therefore separately loaded both Ag and TMO on inert SiO_2_ support before mixing them. The inert SiO_2_ acts as a structural promoter that keeps Ag and TMO spatially isolated. The catalytic performance of these catalysts are summarized in Supplementary Table 10. The low benzyl alcohol conversion (typically below 35%) over ‘Ag/SiO_2_+TMO/SiO_2_' mixtures indirectly validates our assumption that the promoted catalytic performance comes from the direct contact of the metal and the metal oxide. We further confirmed the formation of Ag-TMO interface by charactering the physically-mixed ‘Ag+NiO' catalyst after use. Energy-dispersive X-ray spectroscopy elemental mapping of the spent catalyst shows the attachment of the Ag domain to the Ni domain (Supplementary Fig. 30A). The lattice fringes depicted in the corresponding HRTEM images (Supplementary Fig. 30B) demonstrate that the two crystal domains are chemically bonded together.

### Inverse-interface design leading to more stable catalyst

Having understood the catalytic nature of gas-phase alcohol oxidation over metal/oxide systems, we realize that these insights might enable us to design better catalytic materials with long-term stability. Small metal nanoparticles supported on bulk metal oxides often suffer particle sintering in exothermic or high temperature catalytic processes, leading to fast deactivation of the catalysts^27^. Particular to gas-phase alcohol oxidation, the long-term stability still remains a challenging task^3^,^4^,^5^,^6^. The tendency of particle sintering is usually associated with the melting point of the material. It is worth mentioning two points here: (i) the bulk melting points of metal oxides are generally higher than metals; and (ii) the melting points of nanoparticles are size-dependent; the smaller the size is, the lower the melting point is^28^. In this context, the stability of the nano-metal/micro-oxide catalyst and the inverse counterpart (nano-oxide/micro-metal) must be drastically different, although their catalytic activities are comparable. Taking the Ag–Cu_2_O interface as an example, the benzyl alcohol conversion starts declining within 2 h over nano-Ag/micro-Cu_2_O, but it stays quite stable (>90%) for at least 230 h using the inverse nano-Cu_2_O/micro-Ag catalyst (Supplementary Fig. 31). Nano-Ag supported on micro-Cu_2_O showed serious particle sintering after the stability test, in sharp contrast to the almost unaffected size distribution of nano-Cu_2_O supported on micro-Ag (Supplementary Fig. 32). The long-term stability of the inverse catalyst design was also confirmed by nano-CoO/micro-Ag (Supplementary Fig. 33). In addition, the deactivated nano-Ag/micro-CoO can restore the initial activity (conversion: 91%; selectivity: 99%) simply by post-doping CoO nanoparticles on the surface to reconstruct sufficient Ag–CoO interface. This regeneration strategy is of great potential in recovering other thermally deactivated catalysts, such as three-way catalyst in catalytic converters and the catalyst for removing volatile organic compounds.

### Selective oxidation of other primary alcohols

The effectiveness of metal-oxide interface in benzyl alcohol oxidation has been extended to straight-chain, benzylic and polynary (1,2-propanediol) alcohols (Table 1) using inverse nano-CoO/micro-Ag. 1-phenylethanol is oxidized to acetophenone at a high conversion of 97% at 270 °C, and the primary linear octanol alcohol (1-ol) conversion is 67%. Cyclohexanol is selectively oxidized to cyclohexanone (a key raw material for many useful chemicals, such as caprolactam for nylon 6 and adipic acid for nylon 66) at a conversion of 79% with a selectivity of 95% at 280 °C. Furthermore, alcohols containing two hydroxyl groups, such as 1,2-propylene glycol that is favourable to form hydroxylketone rather than methyl glyoxal, could also be selectively oxidized to the target product (methyl glyoxal) with a selectivity of 72% at a conversion of 94%.

### Rational design of a practical monolithic catalyst

**Figure 5:**
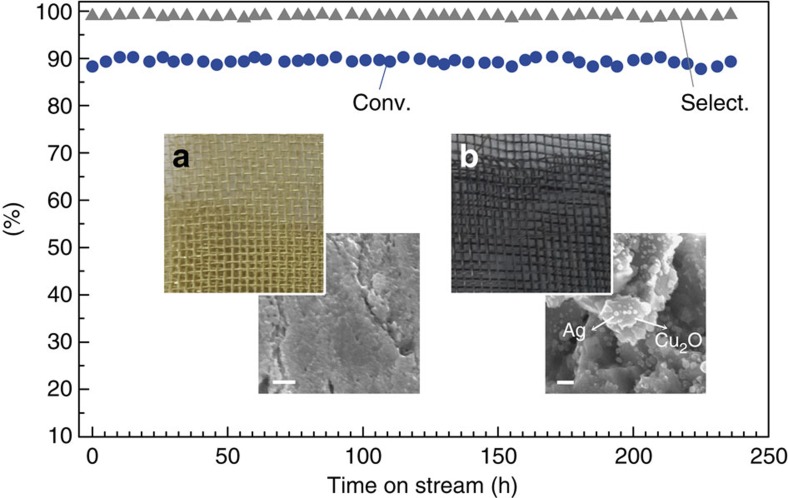
Monolithic catalyst with improved stability on basis of inverse interface design on Cu gauze. The composition of the monolithic catalyst is Cu_2_O/Ag/Cu-gauze (3 wt% Cu_2_O loading and 10 wt% Ag loading). Digital photos and scanning electron microscopic images of Cu gauze (**a**) as well as Cu_2_O/Ag/Cu-gauze catalyst (**b**). Stability test conditions: catalyst (1 g), benzyl alcohol (4 g h^−1^), air (60 ml min^−1^), 0.1 MPa, 250 °C. The monolithic catalyst also exhibits superior properties in terms of space velocity, heat/mass transfer, and pressure drop in catalyst bed (Supplementary Fig. 39). Scale bar, 200 nm.

Guided by the interfacial effects revealed here, we designed a monolithic catalyst eligible for future industrial application, that is, Cu_2_O/Ag/Cu-gauze with three-dimensional open structures (Fig. 5). To realize the Ag–Cu_2_O interface on the commercially available Cu-gauze, a thin silver film was coated on the surface of Cu-gauze by silver mirror reaction^29^. Then the copper oxides were deposited on the Ag/Cu-gauze matrix. Scanning electron microscopic images show that the surface of Cu-gauze (Fig. 5a) is coated with Ag and Cu_2_O (Fig. 5b), and X-ray diffraction patterns also exhibit the characteristic peaks of Ag and Cu_2_O (Supplementary Fig. 34). The gas-phase selective oxidation of benzyl alcohol was subsequently conducted. Over the Cu_2_O/Ag/Cu-gauze, benzyl alcohol conversion is 95% at 300 °C and 91% at 260 °C. In contrast, the Ag/Cu-gauze and Cu_2_O/Cu-gauze show much lower benzyl alcohol conversions at the corresponding reaction temperature (Supplementary Fig. 35). *Ex situ* characterizations including X-ray diffraction, N_2_ physical adsorption, and XPS were performed on the monolithic catalyst during the course of on-stream running. As shown in Supplementary Figs 36–38, the phase compositions, surface areas, as well as the oxidation states of Cu and Ag in the spent catalyst stay almost unchanged compared with the fresh one. The outstanding structural stability of the monolithic catalyst leads to exceptionally steady performance. No obvious decay in activity and selectivity was observed upon 250 °C for at least 230 h (Fig. 5). Technically, the monolithic metal-oxide catalysts built on Cu-gauze have many benefits including a wide range of space velocity (4–15 h^−1^), more efficient heat/mass transfer with lower temperature rising and pressure drop in catalyst bed as shown in Supplementary Fig. 39 (ref. 30), as well as better interphase contact. These benefits can be potentially extended to other related industrial processes, such as vehicle emissions control and volatile organic compounds removal.

In summary, a variety of metal/oxide combinations have been demonstrated as highly active and selective catalysts for the gas-phase aerobic oxidation of benzyl alcohol and other primary alcohols. The metal-oxide interface is identified as the active sites, on the basis of STM measurements on FeO/Pt(111) and Cu_2_O/Ag(111) model catalysts as well as DFT calculations. The boundary oxygen, which comes from facile O_2_ dissociation at the interfacial sites, facilitates the adsorption of benzyl alcohol and O–H bond cleavage through a strong binding with the hydrogen of hydroxyl group. A proper oxygen binding strength plays a crucial role in a complete catalytic circle and is proposed to be a general energy descriptor for highly active catalysts. We also prove that the interfacial effects are size insensitive by comparing the three types of interface designs, that is, nanocomposite, nano-metal/micro-oxide, and the inverse nano-oxide/micro-metal. We point out that the inverse nano-oxide/micro-metal catalyst offers a promising solution to particle sintering problem which usually accounts for catalyst deactivation. With these fundamental insights, we further develop a practical monolithic catalyst on Cu gauze, which exhibits exceptional long-term stability, wide space velocity, efficient heat/mass transfer, and low pressure drop. This study highlights the importance of determining the active sites for the rational development of better catalysts beyond laboratory interest.

## Methods

### Characterization

Pt NPs, Pd NPs, PdCu nano-alloy, Ag-Cu_2_O nanocomposite, Ag, Cu_2_O, transition metal oxides (TMO) NPs and the catalyst samples were characterized by powder X-ray diffraction (Bruker D8-advance X-ray powder diffractometer (Cu Kα, *λ*=1.5406 Å)), transmission electron microscope (TEM, Hitachi model H-800), HRTEM (recorded by a FEI Tecnai G2 F20 S-Twin high-resolution transmission electron microscope working at 200 kV and a FEI Titan 80–300 transmission electron microscope equipped with a spherical aberration (Cs) corrector for the objective lens working at 300 kV), and scanning electron microscope (JSM-6301F). XPS were recorded on a VG EscaLab 220i-XL spectrometer using a standard Al Kα X-ray source (300 W) and an analyzer pass energy of 20 eV. All binding energies were referenced to the adventitious C1s line at 284.9 eV. Specific surface area was determined from N_2_ adsorption isotherm at −196 °C using standard Brunauer–Emmett–Teller (BET) theory. Before the measurement of N_2_ adsorption, degassing was conducted at 300 °C for 4 h. The catalyst loadings were determined by inductively coupled plasma atomic emission spectrometry (ICP-AES) on a Thermo Scientific iCAP 6300 ICP spectrometer.

### Gas-phase alcohol oxidation test

The gas-phase selective oxidation of alcohols was performed on a fixed-bed quartz tube reactor (700 mm in length and 7 mm of inner diameter) under atmospheric pressure. Catalyst was loaded in the tube reactor. Alcohols were continuously fed into the reactor using a high-performance liquid pump in parallel with air feeding using calibrated mass flow controllers. Weight hourly space velocity was calculated by dividing the weight of the fed alcohols per hour by that of catalyst. The effluent was cooled using an ice-salt bath (−15 °C) to liquefy the condensable vapors for analysis using an HP 5890 gas chromatography-flame ionization detector with a 60 m HP-5 ms capillary column. The gas-phase products, such as H_2_, CO_*x*_ and C1–C3 hydrocarbons, were analysed using an HP-5890 GC with thermal conductivity detector and a 30 m AT-plot 300 capillary column.

### STM experiments

The experiments were carried out in a combined ultrahigh vacuum (UHV) system equipped with Createc low temperature scanning tunnelling microscope (LT-STM), XPS, UPS and the cleaning facilities. The STM and preparation chambers have a base pressures of 4 × 10^–11^ mbar and 6 × 10^–11^ mbar, respectively. The Pt(111) single crystal (Matek) was cleaned by cycles of Ar ion sputtering (1.5 keV, 10 μA) and annealing at 1,200 K. Nano-sized FeO islands were deposited onto Pt(111) by vapor deposition of Fe atoms in an O_2_ atmosphere (P(O_2_)=1 × 10^–7^ Torr) with the temperature of Pt(111) held at 300 K. The as-deposited surface was then annealed in UHV at 500 K, leading to the formation of well-ordered FeO islands. The Ag(111) single crystal (Matek) was cleaned by cycles of Ar ion sputtering (1.5 keV, 10 μA) and annealing at 800 K. Cu_2_O islands were deposited onto Ag(111) by vapor deposition of Cu atoms in an O_2_ atmosphere (P(O_2_)=1 × 10^–7^ Torr) with the temperature of Ag(111) held at 117 K. The as-deposited surface was then annealed in UHV at 350 K, leading to the formation of well-ordered Cu_2_O islands. The sample cleaning, preparation and gas exposure were performed in the preparation chamber and STM imaging were conducted in the LT-STM chamber. STM images were processed and analysed with the SPIP software (Image Metrology, Denmark).

### Data availability

All the relevant data are available from the authors upon request.

## Additional information

**How to cite this article:** Zhao, G. *et al*. Metal/oxide interfacial effects on the selective oxidation of primary alcohols. *Nat. Commun.* **8,** 14039 doi: 10.1038/ncomms14039 (2017).

**Publisher's note:** Springer Nature remains neutral with regard to jurisdictional claims in published maps and institutional affiliations.

## Supplementary Material

Supplementary Information Supplementary Figures, Supplementary Tables, Supplementary Discussion, Supplementary Methods and Supplementary References.

## Figures and Tables

**Table 1:** Oxidation of various alcohols over the nano-CoO/micro-Ag, micro-Ag and nano-CoO*.

**Catalysts**	**Substrate**	**O**_**2**_ **/ol ratio (mol/mol)**	***T*** **(°C)**	**Conversion (%)**	**Selectivity (%)**
Nano-CoO/micro-Ag	1-phenylethanol	0.8	270	97	97
	1-octanol	0.6	300	67	92
	Cyclohexanol	0.6	280	79	95
	1,2-propanediol	1.6	320	94	72
Micro-Ag	1-phenylethanol	0.8	270	31	98
	1-octanol	0.6	300	15	94
	Cyclohexanol	0.6	280	18	96
	1,2-propanediol	1.6	320	28	77
Nano-CoO	1-phenylethanol	0.8	270	13	99
	1-octanol	0.6	300	9	95
	Cyclohexanol	0.6	280	13	96
	1,2-propanediol	1.6	320	16	81

^*^Reaction conditions: catalyst (0.15 g), WHSV (20 h^−1^), N_2_ (50 ml min^-1^). Flow of O_2_ was determined by the O_2_/ol ratio (mol/mol) of the corresponding reaction.
