# Supplementary material for: Metal/oxide interfacial effects on the selective oxidation of primary alcohols
Source: Nat Commun. 2017 Jan 18;8:14039. doi: 10.1038/ncomms14039 (PMC5253635; doi:10.1038/ncomms14039)
Supplement: Supplementary Information — Supplementary Figures, Supplementary Tables, Supplementary Discussion, Supplementary Methods and Supplementary References. [file ncomms14039-s1.pdf]

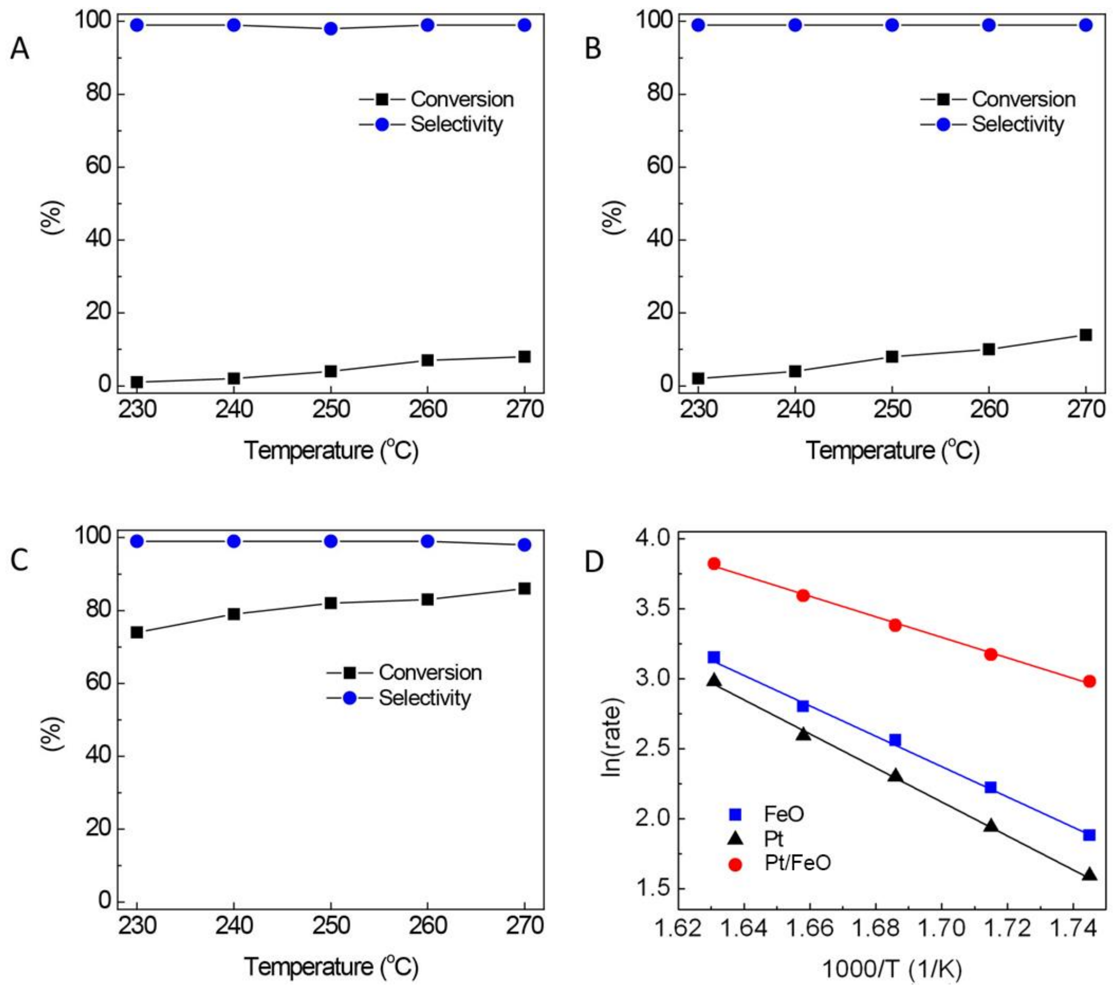

Supplementary Figure 1.

Benzyl alcohol conversion and benzaldehyde selectivity over the catalysts of FeO (**A**), Pt (**B**) and Pt/FeO (**C**) colloidal nanoparticles supported on SiO_2. Reaction conditions: catalyst (0.15 g), Pt or FeO loading (4 wt%), WHSV (20 h^{-1}), air (50 mL/min). (**D**) Apparent activation energies (E_a) over the Pt (102 kJ/mol), FeO (91 kJ/mol) and Pt/FeO (62 kJ/mol) colloidal nanoparticles supported on SiO_2. The kinetic studies were conducted under the conditions that could eliminate the influence of heat/mass diffusion: catalyst (7 mg), Pt or FeO loading (4 wt%), WHSV (50 h^{-1}), O_2 (2 mL/min), N_2 (100 mL/min), SiO_2 support (200 mesh), quartz sand 0.2 g (100 mesh).

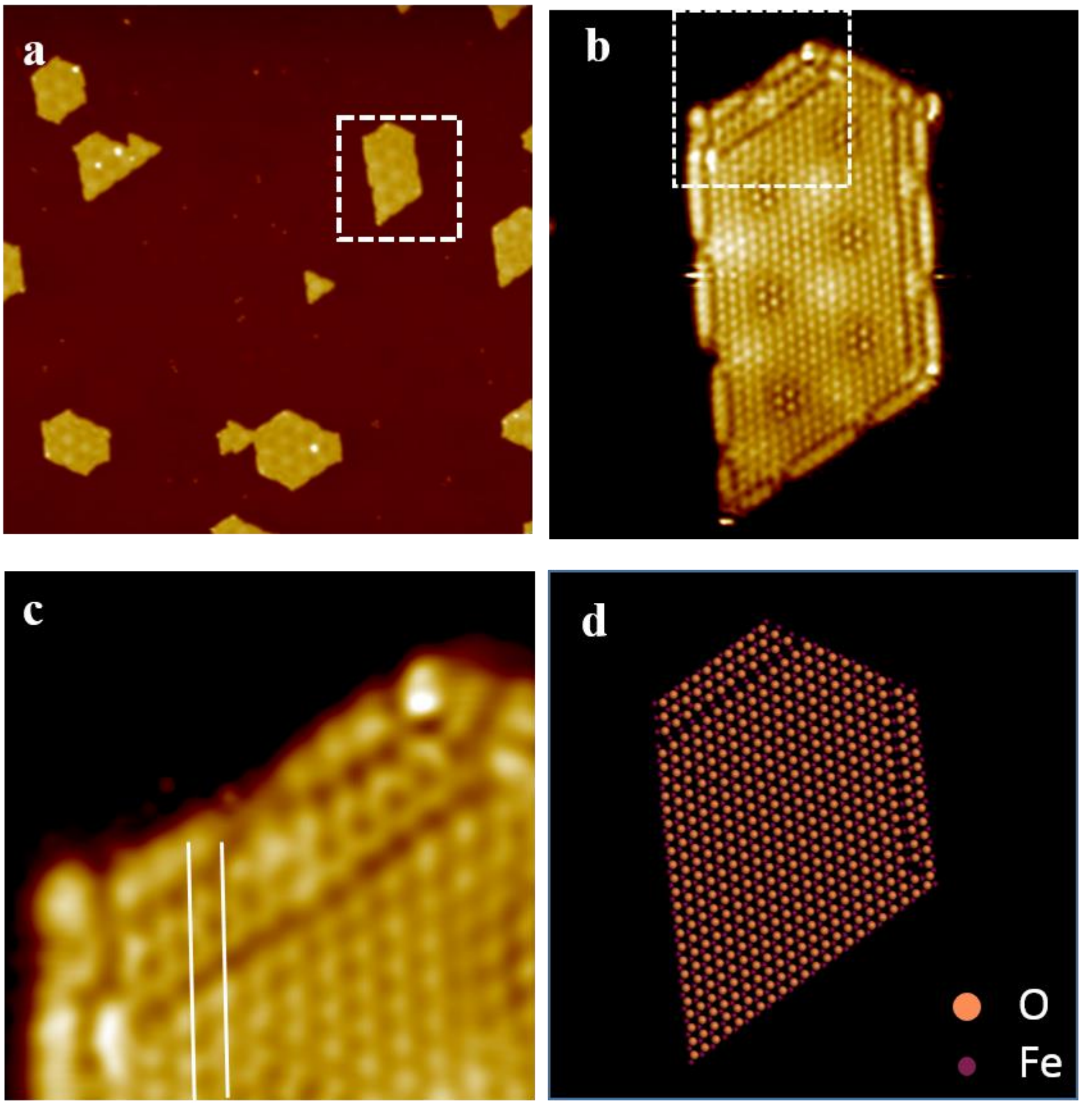

Supplementary Figure 2.

STM images of FeO(111) islands with Fe-terminated steps on Pt(111). (**a**) FeO nanoislands were annealed in UHV at 550 K and expose dominantly Fe-terminated steps. (**b**) The atomic structure of an Fe-terminated FeO nanoisland marked in (a). (**c**) The magnification of the white square in (b). O atoms were resolved as bright protrusions in the STM image. (**d**) The structural model illustrating the structure of Fe-terminated FeO island. Image sizes: (a) 57 nm x 57 nm; (b) 13 nm x 13 nm; (c) 4 nm x 4 nm.

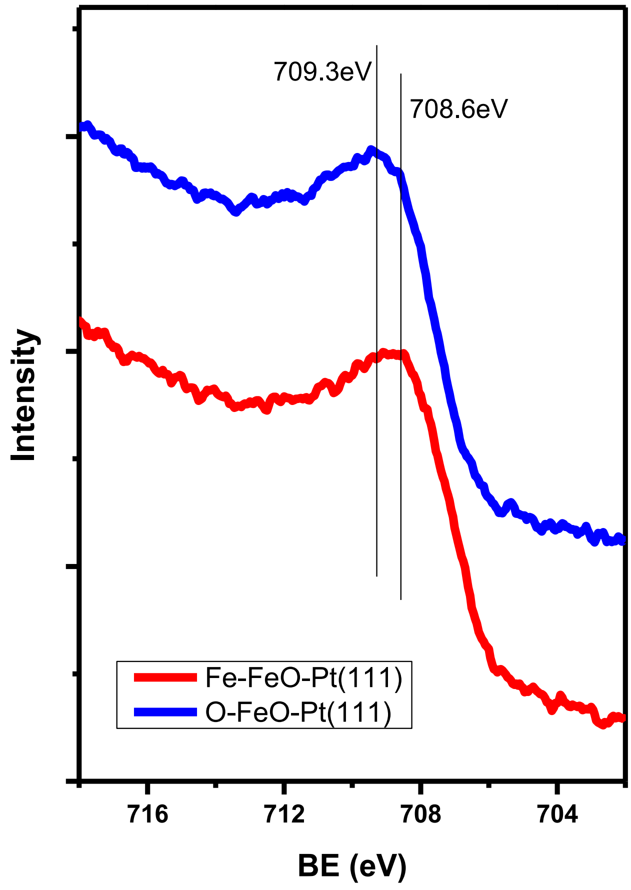

Supplementary Figure 3.

Fe $2p_{3/2}$ spectra of the Fe-FeO-Pt(111) surface (FeO islands with dominantly Fe-terminated edges) and the O-FeO-Pt(111) surface (FeO islands with dominantly O-terminated edges) measured by XPS.

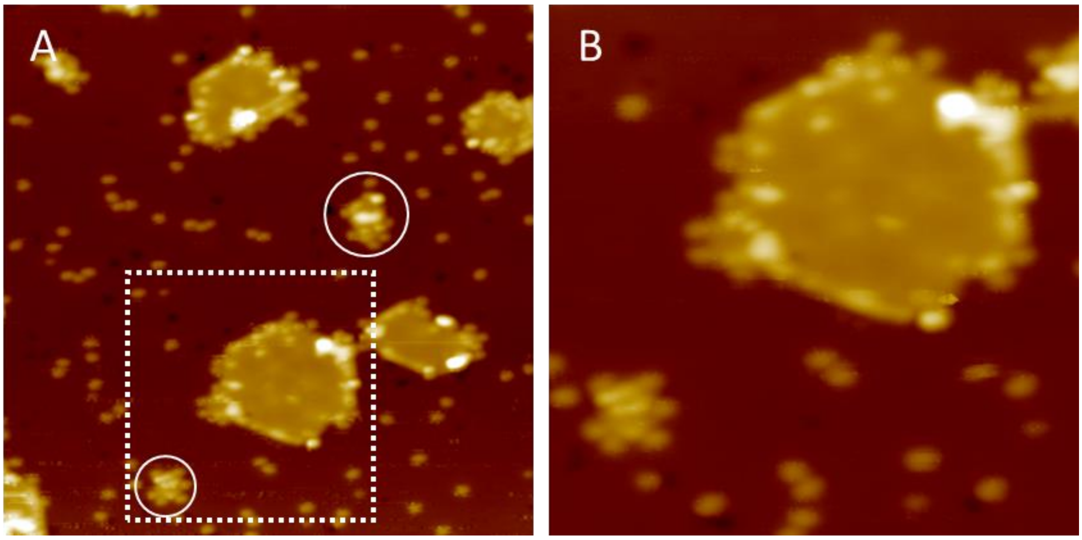

Supplementary Figure 4.

(**A**) STM image of O-FeO(111)/Pt(111) after exposure of ~0.4 L (1 L = 1.0 × 10^{-6} Torr·s) benzyl alcohol at 300 K. (**B**) Magnified STM image of the nanoislands marked by white dashed square in (**A**). Image sizes: (**A**) 34 nm × 34 nm, (**B**) 16 nm × 16 nm.

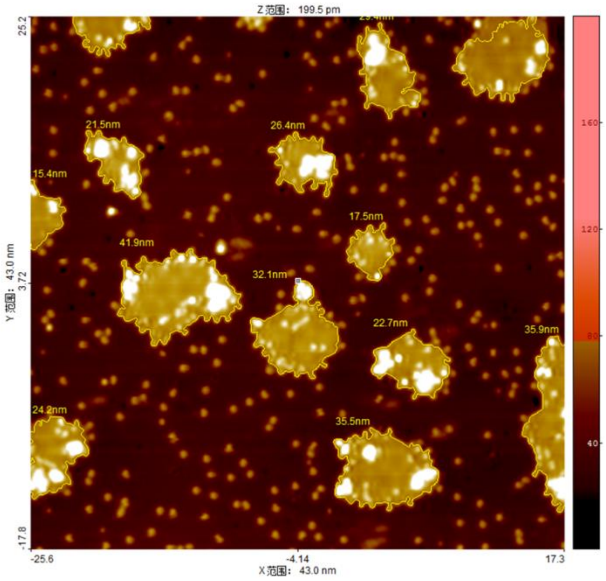

Supplementary Figure 5.

Calculation of benzyl alcohol density adsorbed at Pt(111) surface and FeO/Pt interface after exposure ~1 L benzyl alcohol at 300 K, followed by flash annealing to 350 K to remove trace diffusive adsorbates. Image sizes: 43 nm × 43 nm.

Note:

Over the interfacial sites: The total length of FeO islands is 357.9457 nm and the distance of O-O at the perimeter is 0.31 nm, so the sites number is 1154.66 (357.9457/0.31). The number of the adsorbed benzyl alcohol is 152. **Molecule per site is 0.132.**

Over the Pt sites: The total area is 1849 nm^2 and the area of FeO islands is 301.0585 nm^2, so the Pt area is 1548 nm^2. The diameter of Pt atom is 0.2775 nm, so the occupied area of one Pt atom is 0.06668 (√3d2/2) nm^2, and the number of Pt atoms is 23215. **Molecule per Pt atom is 0.0096 (234/23215).**

Over the Pt-O sites: Assuming that the adsorbed oxygen is the sites for benzyl alcohol adsorption, the number of the adsorption sites for benzyl alcohol is 5803 based on the mode of Pt(2×2)-O. **Molecule per O atom is 234/5803 = 0.040.**

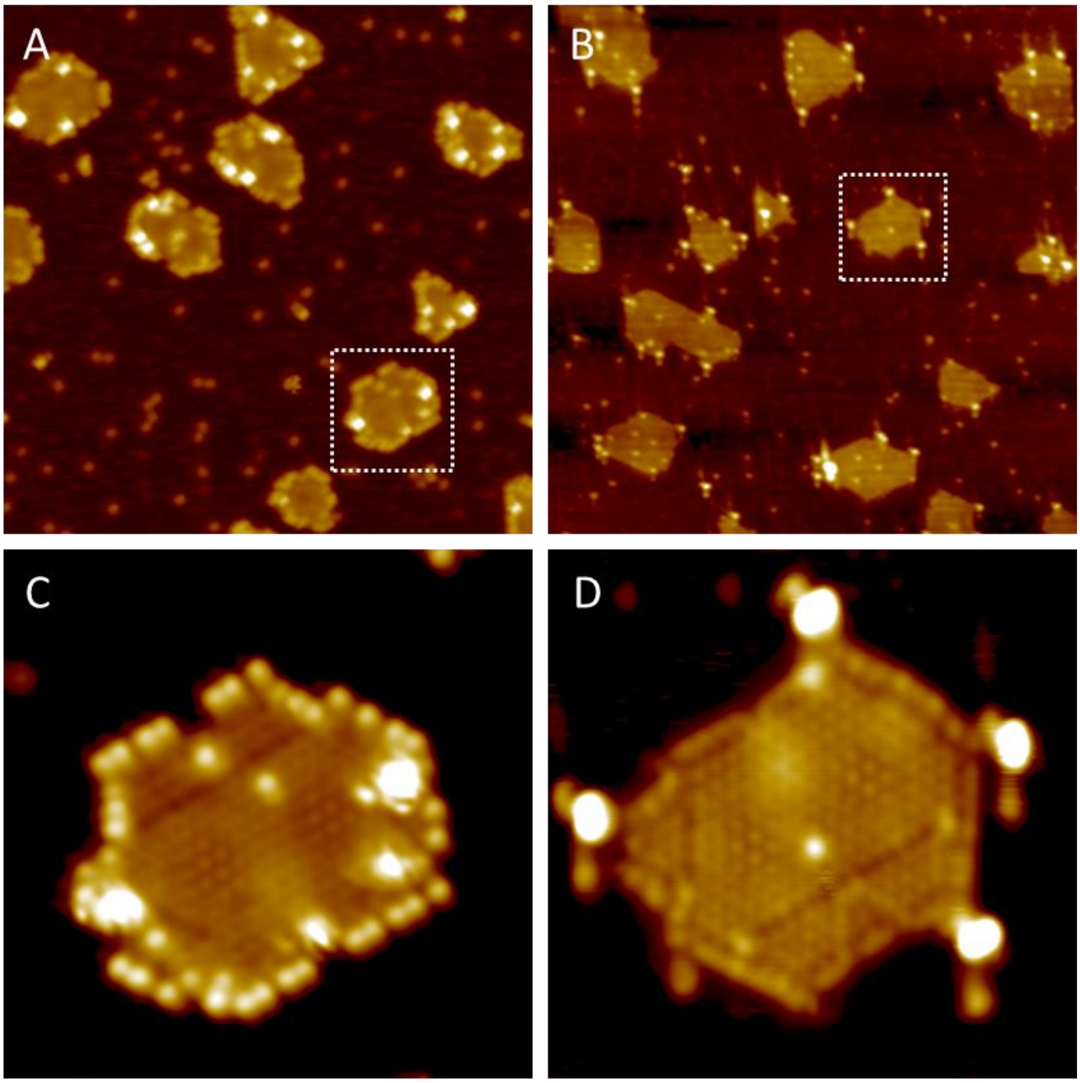

Supplementary Figure 6.

STM images of FeO islands with Fe-terminated steps on Pt(111). (**A**) After the exposure of ~1 L benzyl alcohol at 300 K. (**B**) The surface of (**A**) after flash annealing to 350 K. (**C**) The atomic structure of the FeO island marked by white square in (**A**), showing the adsorption of benzyl alcohol. (**D**) The atomic structure of the FeO island marked in (**B**), showing the desorption of benzyl alcohol. Image sizes: (**A**) 35 nm × 35 nm, (**B**) 47 nm × 47 nm, (**C**) 8.5 nm × 8.5 nm, (**D**) 8.5 nm × 8.5 nm.

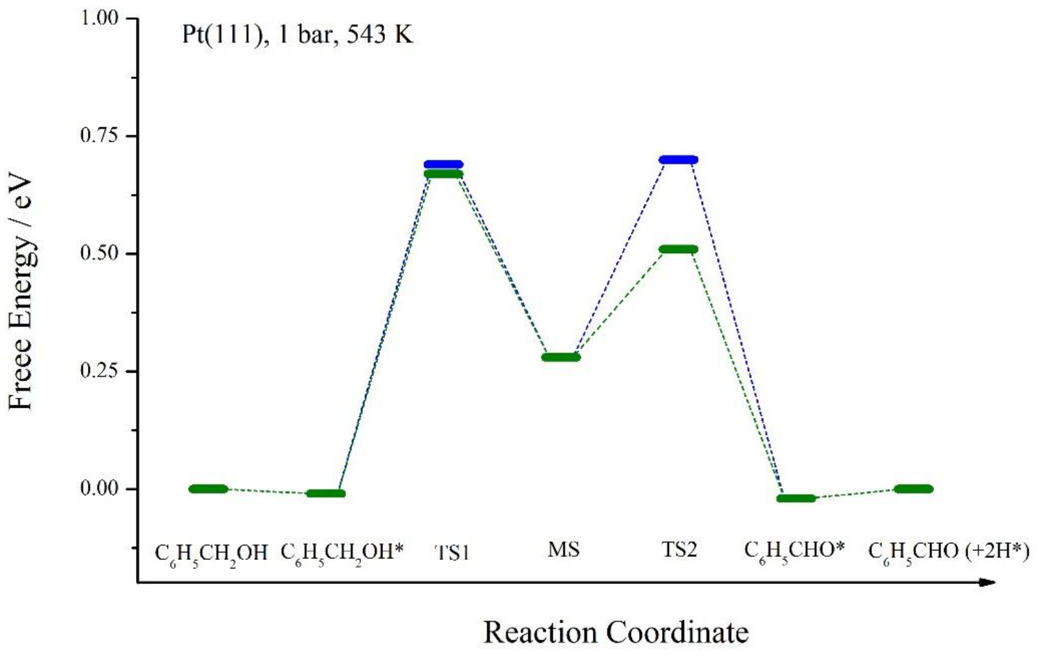

Supplementary Figure 7.

Free energy profiles of benzaldehyde formation on Pt(111). Blue line: pathway 1; green line: pathway 2.

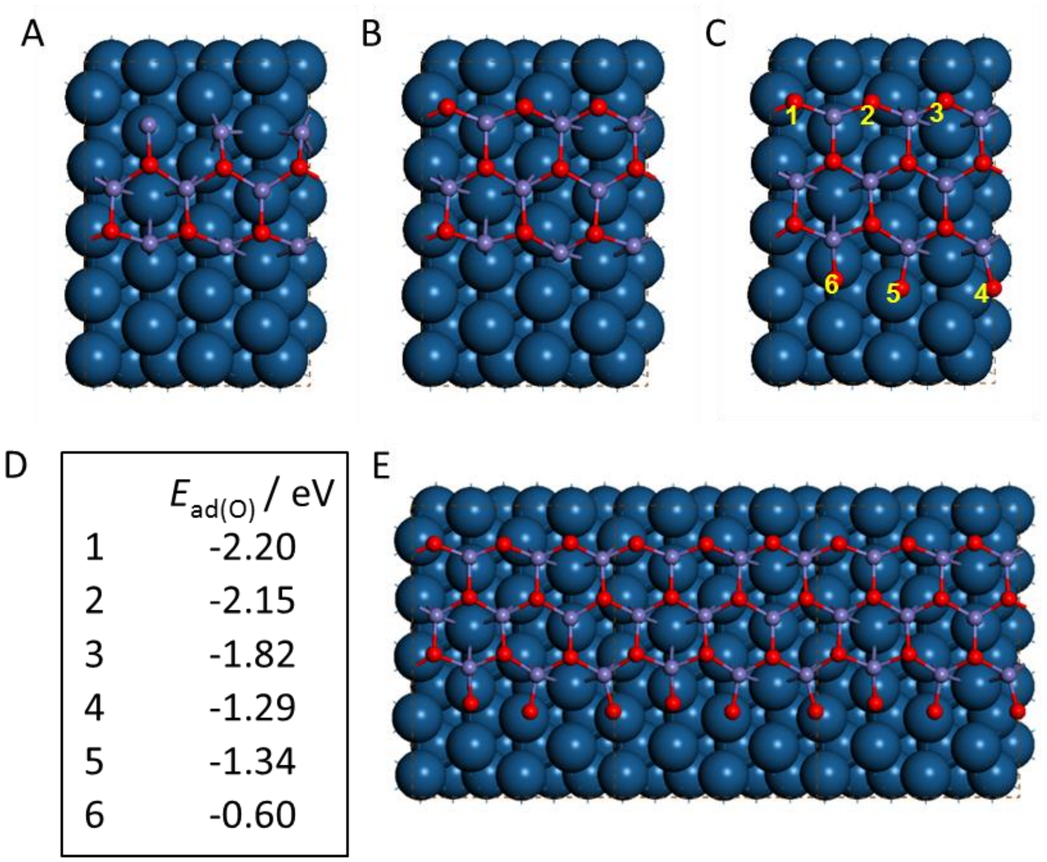

Supplementary Figure 8.

(**A**) and (**B**) Fe-terminated FeO/Pt(111) surfaces. (**C**) O-terminated FeO/Pt(111) surface. (**D**) Oxygen adsorption energies for the formation of O-terminated FeO/Pt(111) surface (the numbers are shown in Supplementary Fig. 8C). (**E**) O-terminated FeO ribbon on Pt(111). Blue balls represent Pt atoms, purple for Fe and red for O.

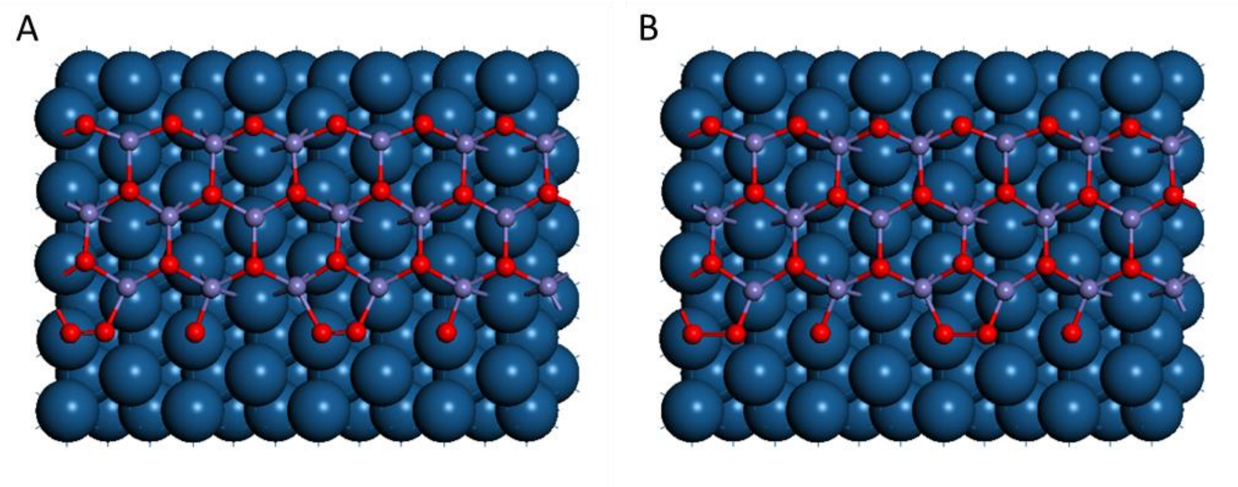

Supplementary Figure 9.

Adsorption and dissociation of molecular oxygen at coordinatively unsaturated Fe sites. (**A**) Adsorption of oxygen (O-O distance: 1.43 Å, adsorption energy: -1.27 eV at -273 ^{o}C). (**B**) Transition state of oxygen dissociation (O-O distance: 1.77 Å, activation energy: 0.34 eV, ΔE = -0.66 eV). Blue balls represent Pt atoms, purple for Fe and red for O.

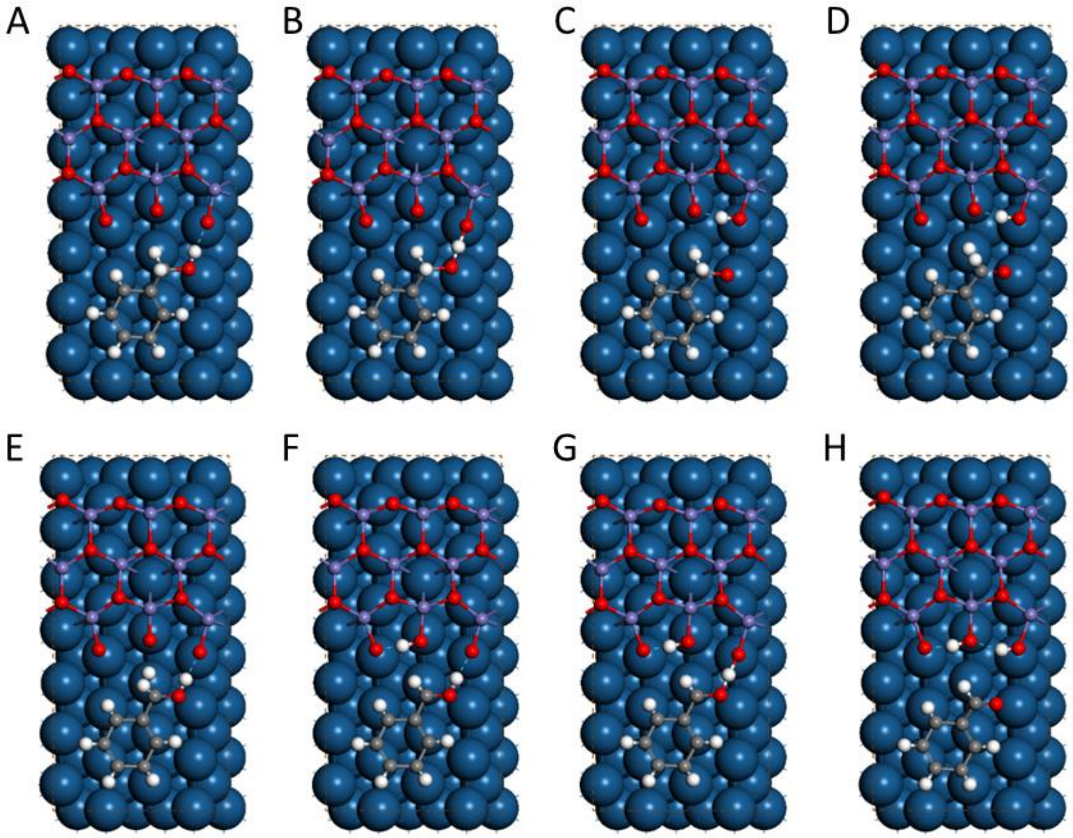

I

Structures		d_{OH_1} [a]	d_{OH_2} [b]	$d_{C\text{-}H}$
Pathway 1	TS1 (fig. S10B)	1.21	1.26	N/A
	TS2 (fig. S10D)	N/A	N/A	1.59
Pathway 2	TS1' (fig. S10E)	N/A	N/A	1.42
	TS2' (fig. S10G)	1.20	1.27	N/A

Supplementary Figure 10.

Structures of each state in benzaldehyde formation at the FeO/Pt(111) interface. (**A**) Configurations of $C_6H_5CH_2OH$ adsorption (strongly exothermic by 3.62 eV). In pathway 1, the transition state of O-H cleavage (**B**); the intermediate state (**C**); and the transition state of C-H cleavage (**D**). In pathway 2, the transition state of C-H cleavage (**E**); the intermediate state (**F**); and the transition state of O-H cleavage (**G**). (**H**) Configurations of C_6H_5OH adsorption. Blue balls represent Pt atoms, purple for Fe, grey for C, white for H and red for O. (**I**) Structural details of TSs: (**a**) d_{OH_1} denotes the O-H distance in TS structures in which O bonding with C. (**b**) d_{OH_2} denotes the O-H distance in TS structures in which O bonding with Fe.

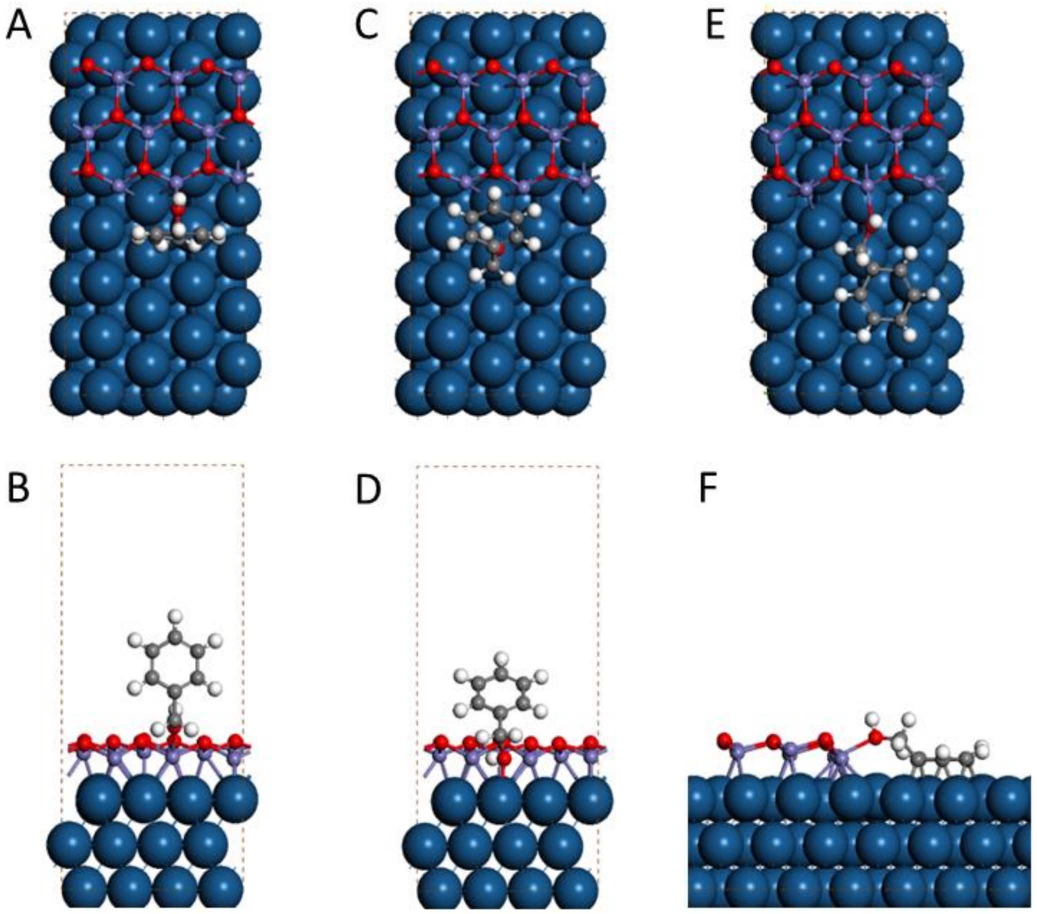

G

Adsorption site	E_{ad} / eV
Fe Site (fig. S11A)	-1.00
Pt Site (fig. S11C)	-0.95
Fe, Pt Site (fig. S11E)	-3.38
Pt(111)	-2.57

Supplementary Figure 11.

Adsorption configurations of $C_6H_5CH_2OH$ at the Fe-FeO/Pt(111) interface. Top view (**A**) and side view (**B**) of $C_6H_5CH_2OH$ adsorption with O bonding with Fe and benzene ring perpendicular to surface. Top view (**C**) and side view (**D**) of $C_6H_5CH_2OH$ adsorption with O bonding with Pt and the benzene ring tilt in vacuum. Top view (**E**) and side view (**F**) of $C_6H_5CH_2OH$ adsorption with O bonding with Fe and benzene ring interact with Pt surface. Blue balls represent Pt atoms, purple for Fe, grey for C, white for H and red for O. (**G**) $C_6H_5CH_2OH$ adsorption energies (-273 ^oC) at Fe-FeO/Pt(111) interface including the effect of van der Waals (vdW) interactions.

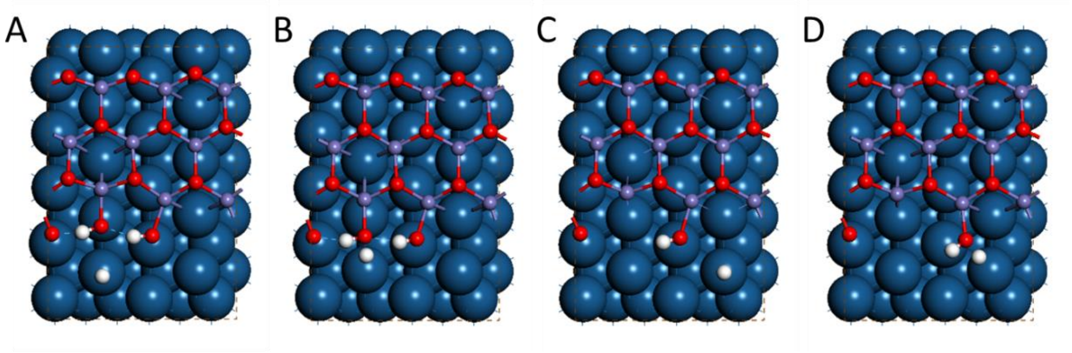

Supplementary Figure 12.

Oxygen vacancy formation at the O-FeO/Pt(111) interface with the aid of dissociated H atoms. (**A**) The formed OH-FeO/Pt interface after O-H and C-H bond cleavage in benzyl alcohol. (**B**) Transition state of first H_2O formation (O-H distance: 1.58 Å). (**C**) First oxygen vacancy formation after H_2O desorption. (**D**) Transition state of second H_2O formation (O-H distance: 1.56 Å). Blue balls represent Pt atoms, purple for Fe, white for H and red for O.

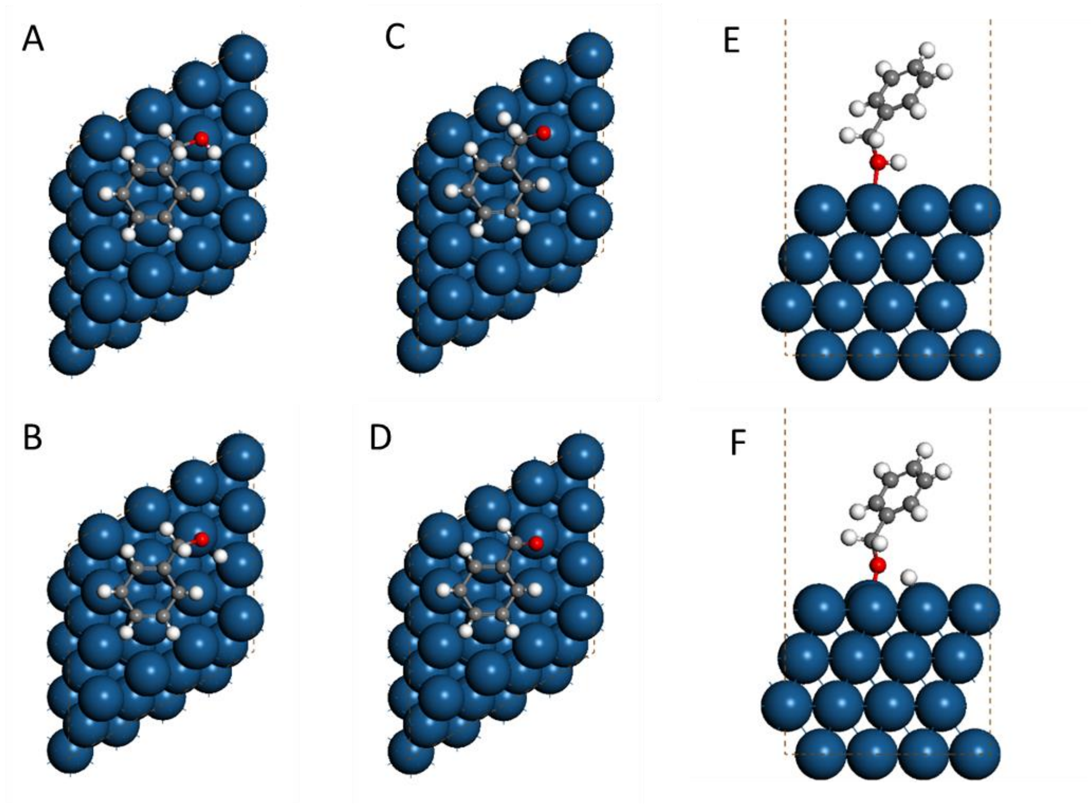

Supplementary Figure 13.

$C_6H_5CH_2OH$ oxidation beginning with O-H bond scission (pathway 1) on Pt(111). (**A**) Adsorption structures of $C_6H_5CH_2OH$. (**B**) and (**C**), Transition state structures of O-H and C-H bond activation, respectively. (**D**) C_6H_5CHO adsorption. (**E**) $C_6H_5CH_2OH$ adsorption with benzene ring tilt in vacuum. (**F**), Transition state structures of O-H bond activation from Supplementary Fig. 13E. Blue balls represent Pt atoms, grey for C, white for H and red for O.

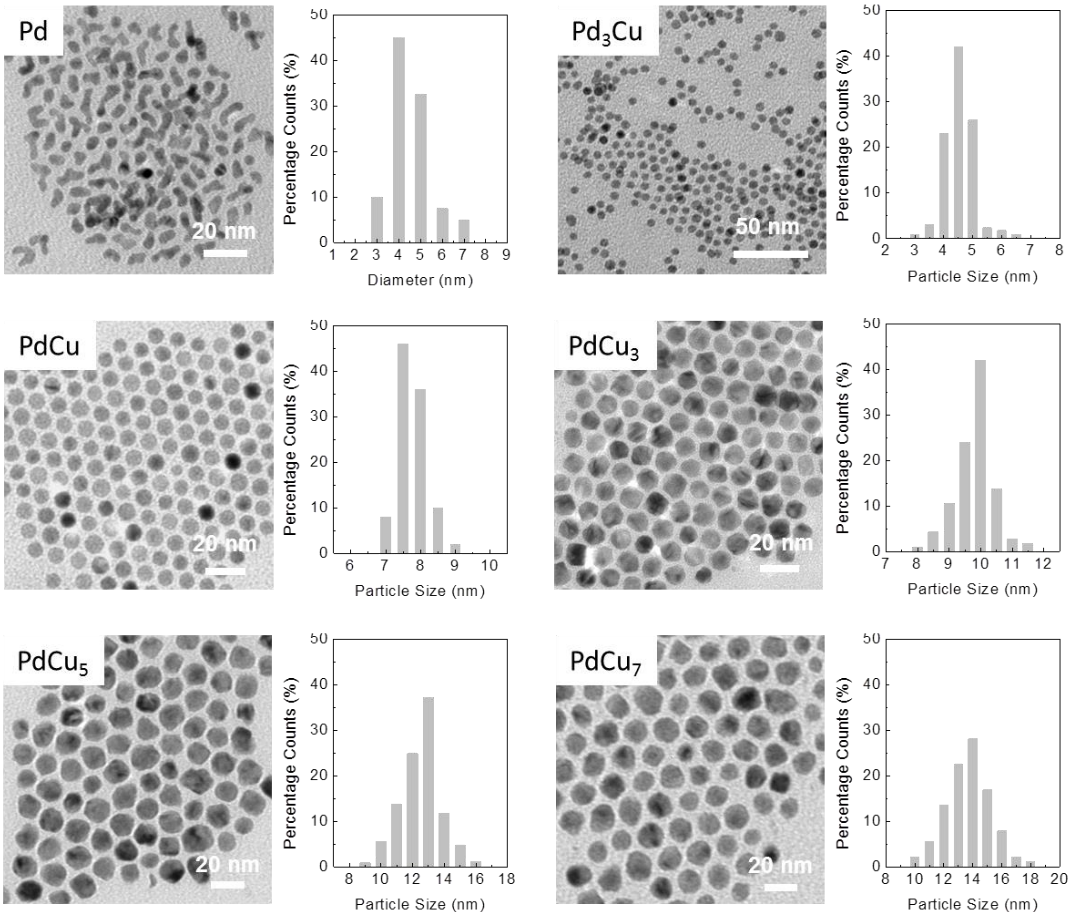

Supplementary Figure 14.

TEM images and the corresponding size distributions of the as-synthesized Pd NPs and Pd-Cu alloy with different Pd/Cu molar ratios.

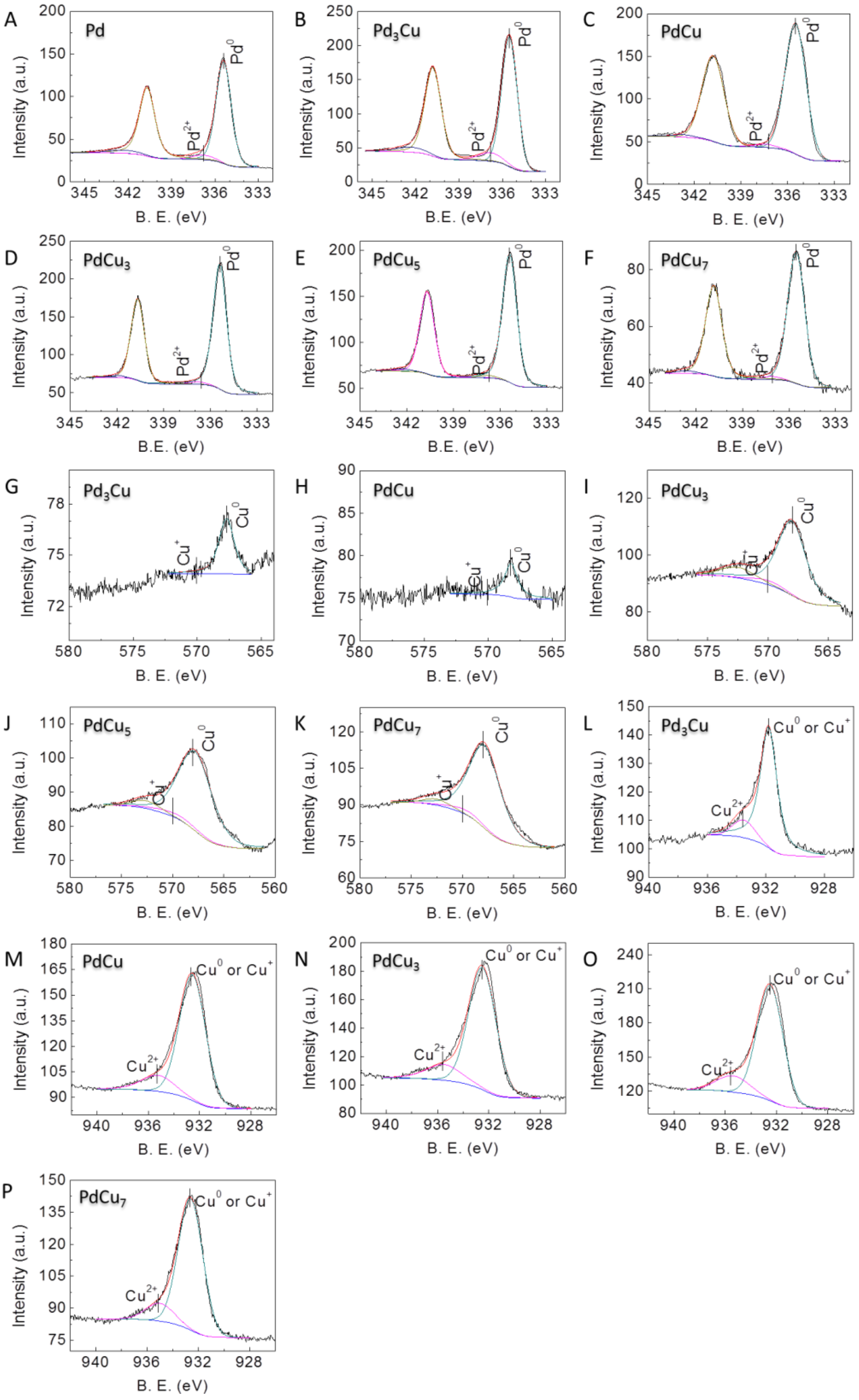

Supplementary Figure 15.

The XPS of Pd 3d peaks (**A−F**) and Cu LMM peaks (**E−K**) and Cu 2p peaks (**L−P**) of the as-synthesized Pd NPs and Pd-Cu alloy.

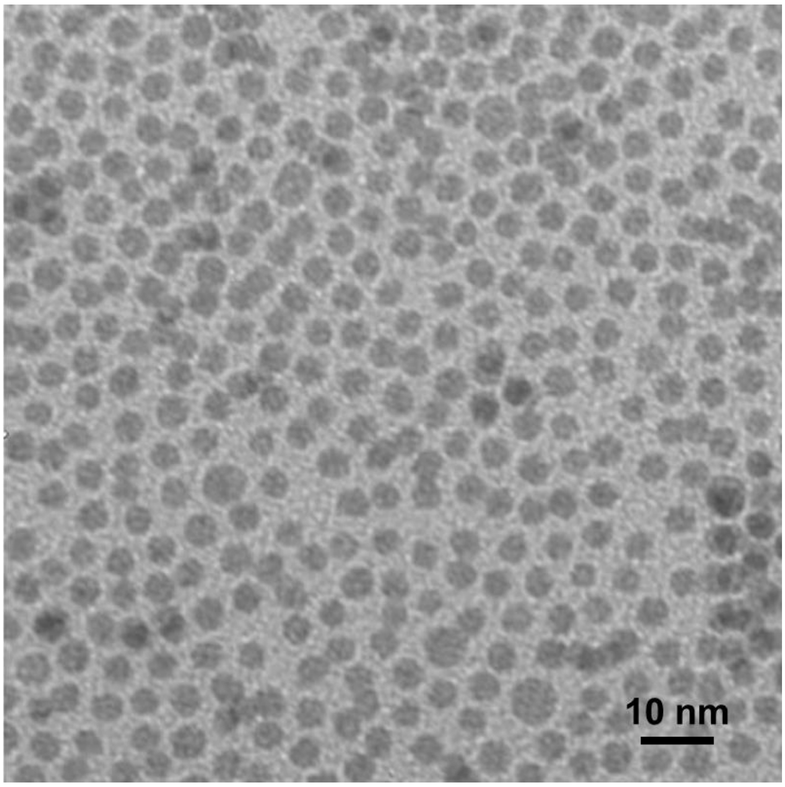

Supplementary Figure 16.

TEM image of the as-synthesized Cu NPs.

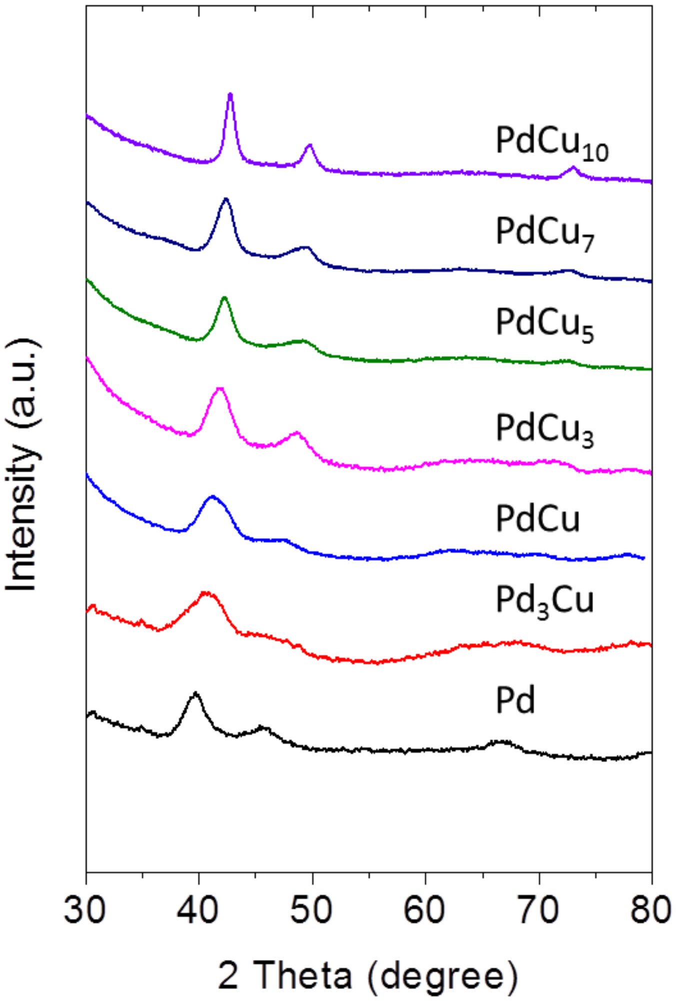

Supplementary Figure 17.

XRD patterns of the as-synthesized Pd NPs and Pd-Cu alloy with different Pd/Cu molar ratios.

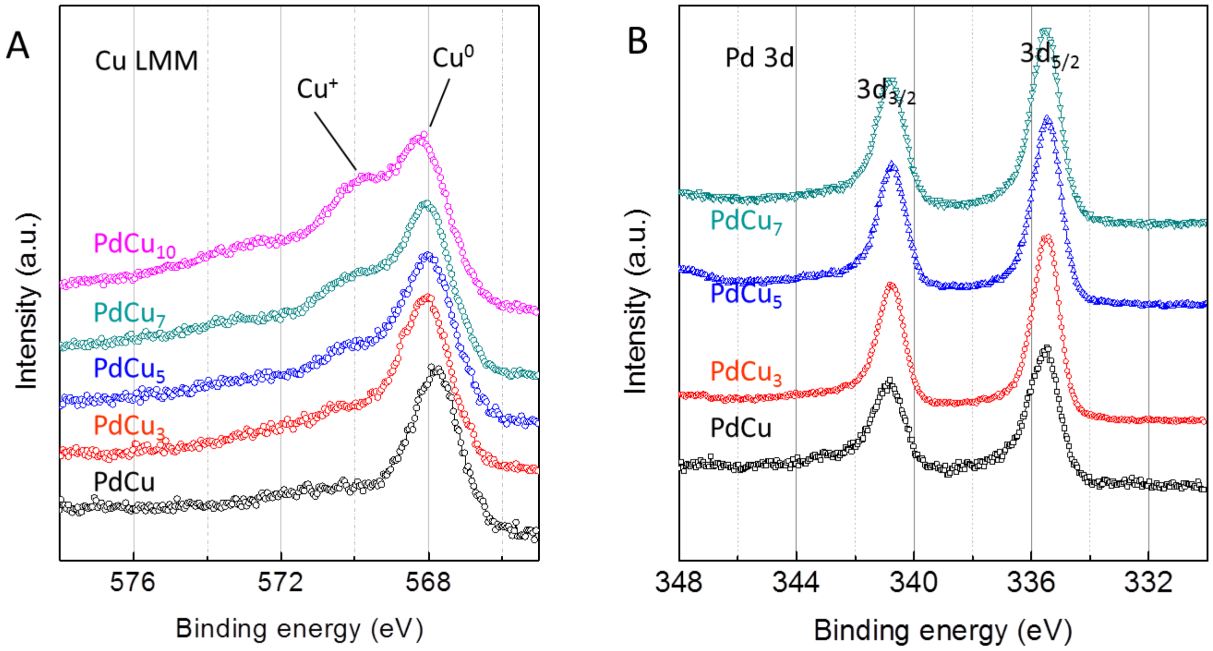

Supplementary Figure 18.

XPS of Cu LMM peaks (**A**) and Pd 3d peaks (**B**) of the used Pd-Cu catalysts.

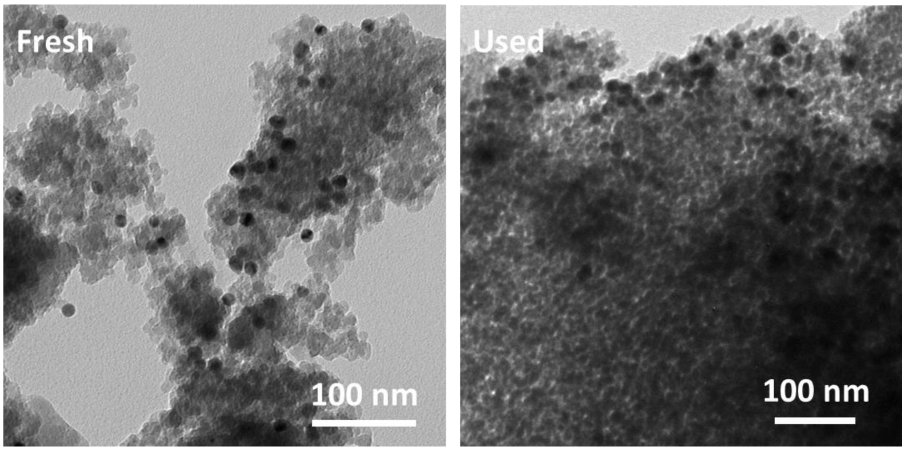

Supplementary Figure 19.

TEM images of the fresh and used $PdCu_7$ catalysts. Catalyst (0.2 g), total metal (Pd and Cu) loading (4 wt%), 270 ^{o}C, WHSV (weight hourly space velocity, 20 h^{-1}), air (60 mL/min), SiO_2 support (200 mesh).

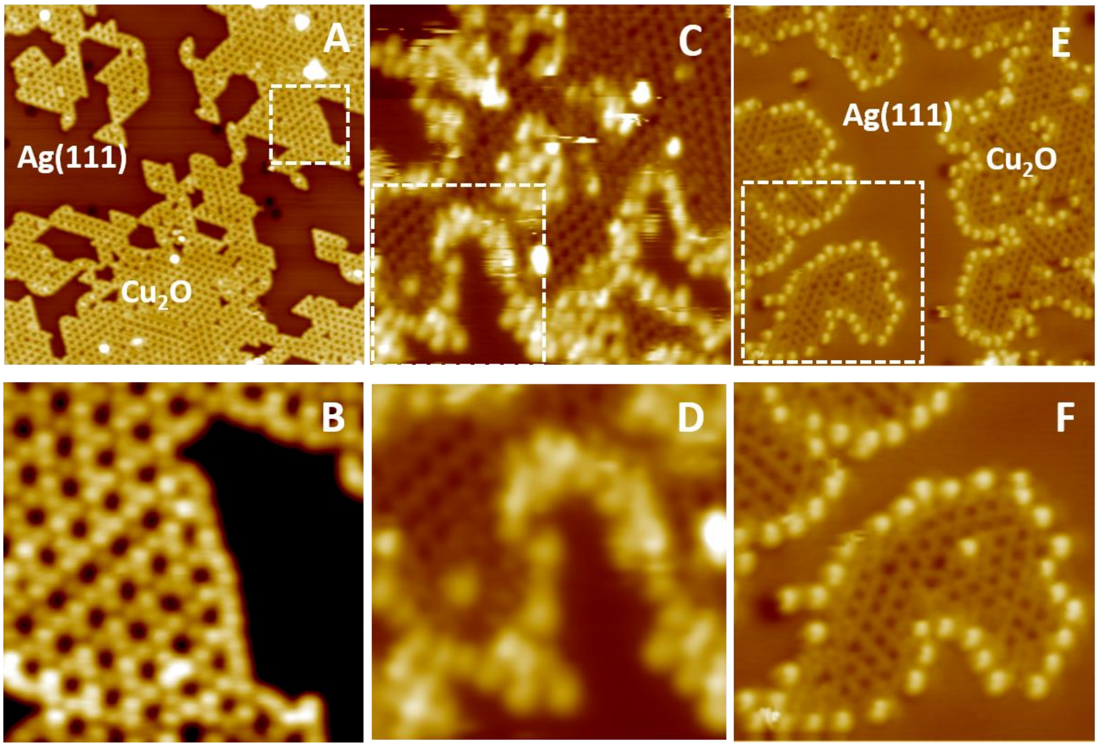

Supplementary Figure 20.

STM images of benzaldehyde formation at the Cu_2O/Ag(111) interface. (**A**) Cu_2O(111) islands grown on the Ag(111) substrate. (**B**) The atomic structure of Cu_2O(111) island marked in (A). (**C**) Benzyl alcohol adsorbed at the Cu_2O/Ag(111) interface at 115 K. (**D**) The magnification of the marked area in (C). (**E**) The surface of (C) after flash annealing to 300 K. (**F**) The magnification of the marked area in (E). Images sizes and scanning conditions: (A) 27 × 27 nm^2, V_s= -2.0 V, I_t= 0.1 nA; (B) 5 × 5 nm^2, V_s= -2.0 V, I_t= 0.1 nA; (C) 19 × 19 nm^2, V_s= -2.2 V, I_t= 0.1 nA; (D) 7 × 7 nm^2, V_s= -2.2 V, I_t= 0.1 nA; (E) 22 × 22 nm^2, V_s= 0.2 V, I_t= 0.6 nA; (F) 10 × 10 nm^2, V_s= 0.2 V, I_t= 0.6 nA.

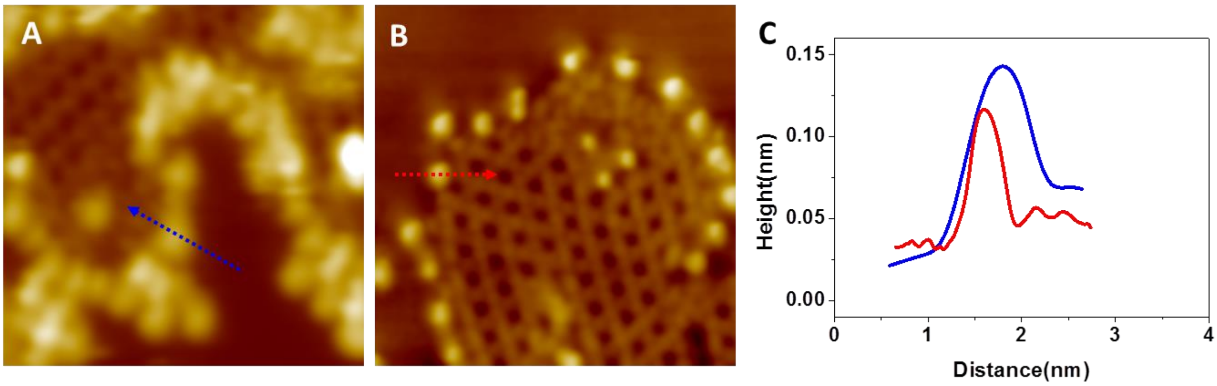

Supplementary Figure 21.

Different appearances of adsorbed species at the Cu_2O/Ag(111) interface before and after UHV annealing. (**A**) Benzyl alcohol adsorbed at the Cu_2O/Ag(111) interface at 115 K; (**B**) The surface of (A) after flash annealing to 300 K. (**C**) Line profiles of adsorbed species: blue line for the species marked in (A) and the red line for the species in (B). Both images are 7 nm x 7 nm in size.

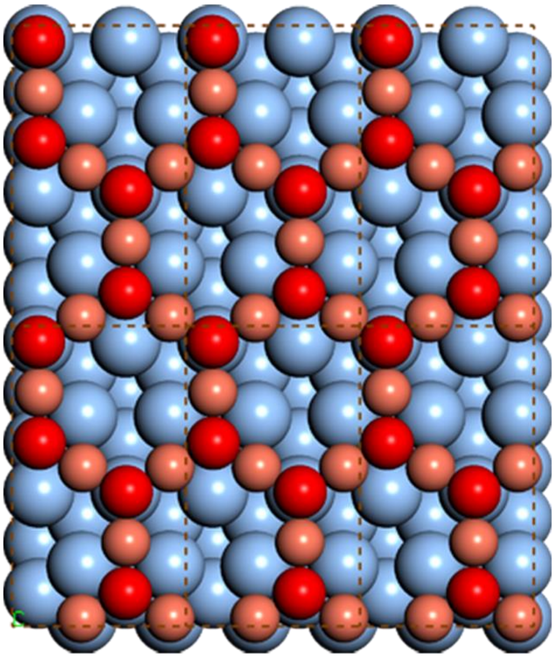

Supplementary Figure 22.

Structures of optimized Cu_6O_4 layer on Ag(111). Light-blue balls represent for atoms of Ag, orange for Cu, and red for O. The lattice spacing of CuO_x is 0.59 nm, agreeing well with the STM result (0.60 nm).

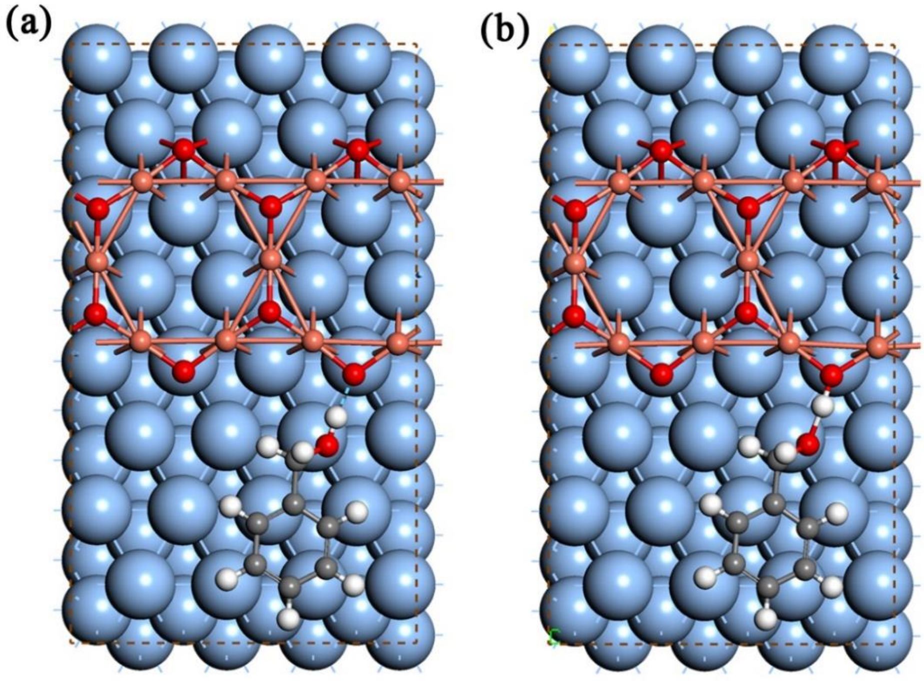

Supplementary Figure 23.

Optimized structures of O-H bond activation of benzyl alcohol at the CuO_x/Ag(111) interface. (**a**) Initial state; (**b**) transition state. Light-blue balls represent for Ag, orange for Cu, red for O, white for H and grey for C.

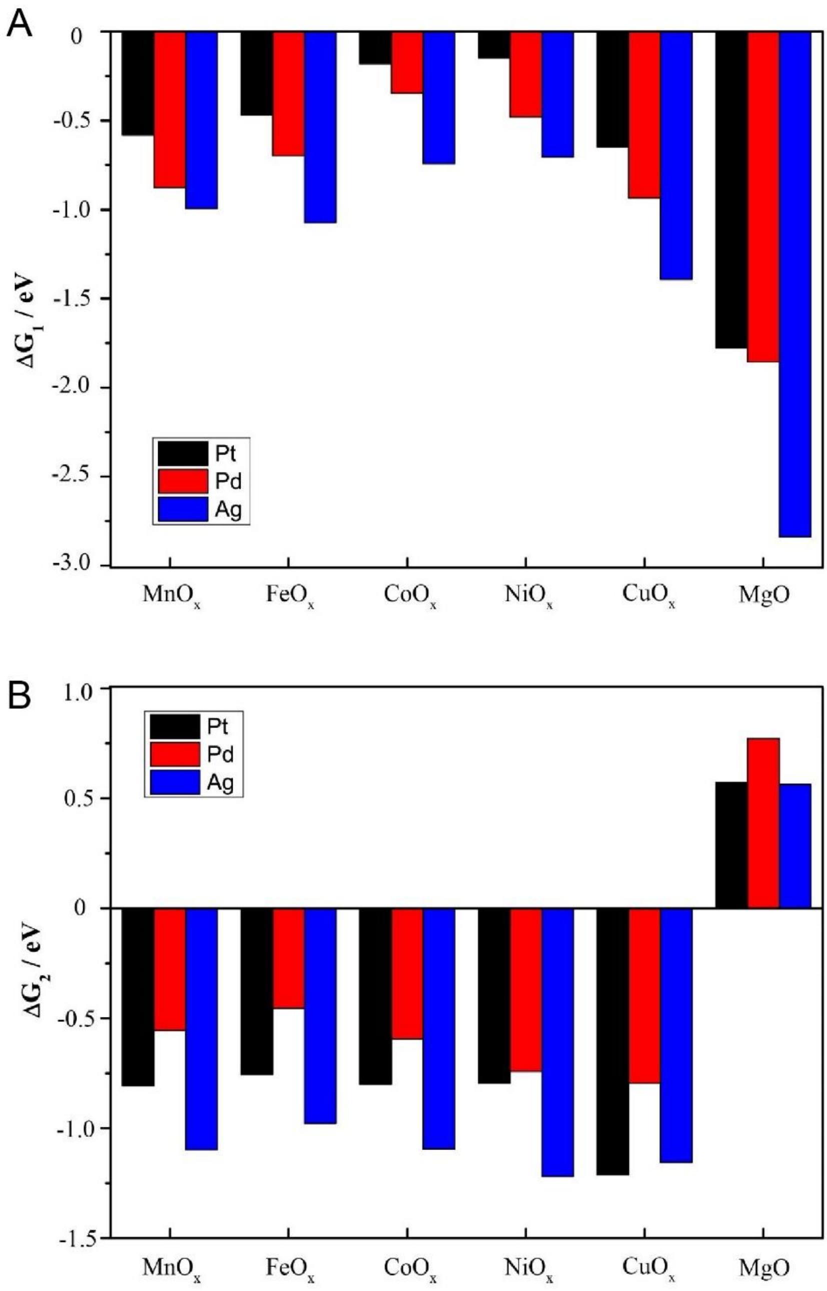

Supplementary Figure 24.

(**A**) Free energy changes of oxygen dissociative adsorption (1/2 O_2 + * → O*) at the edge of Pt, Pd, Ag-supported MO_x ribbon (M = Mn, Fe, Co, Ni, Cu, and Mg) under the reaction conditions (543 K). (**B**) Free energy changes of OH removal reaction (OH* + H* → H_2O + 2*) at the edge of Pt, Pd, Ag-supported MO_x ribbon (M = Mn, Fe, Co, Ni, Cu, and Mg) under the reaction conditions (543 K).

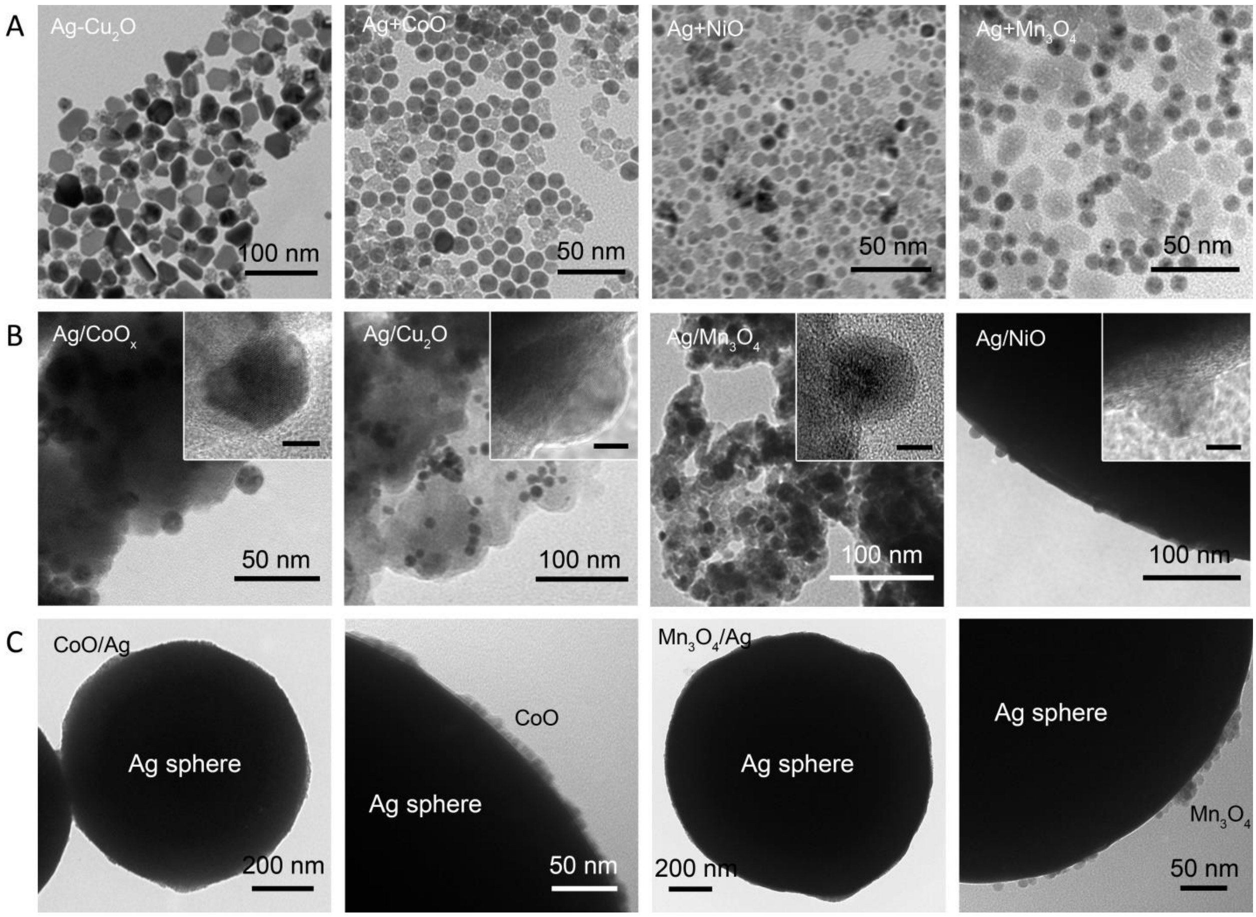

Supplementary Figure 25.

TEM images of (**A**) Ag-TMO nanocomposites, (**B**) nano-TMO/micro-Ag and (**C**) nano-Ag/micro-TMO. Note: the average size of micro-Ag is 1000 nm.

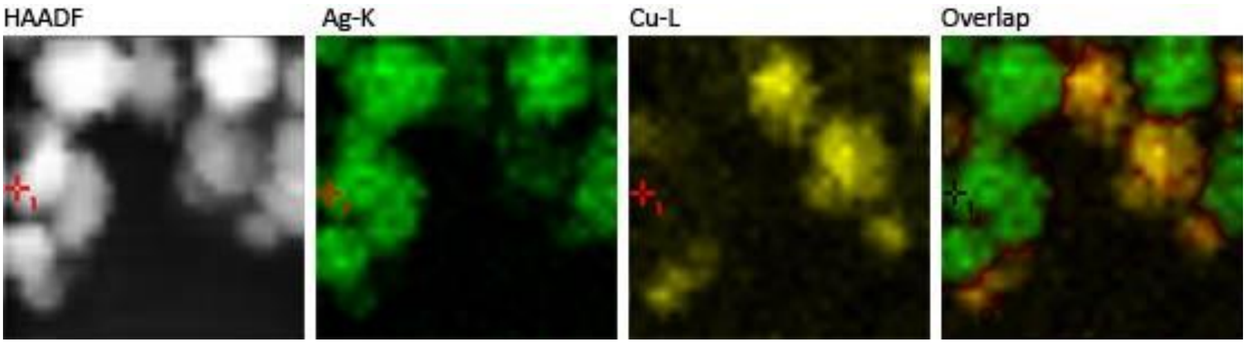

Supplementary Figure 26.

HAADF-STEM image and the corresponding EDS mapping (Ag-K, Cu-L and overlap) of the Ag-Cu_2O nanocomposites.

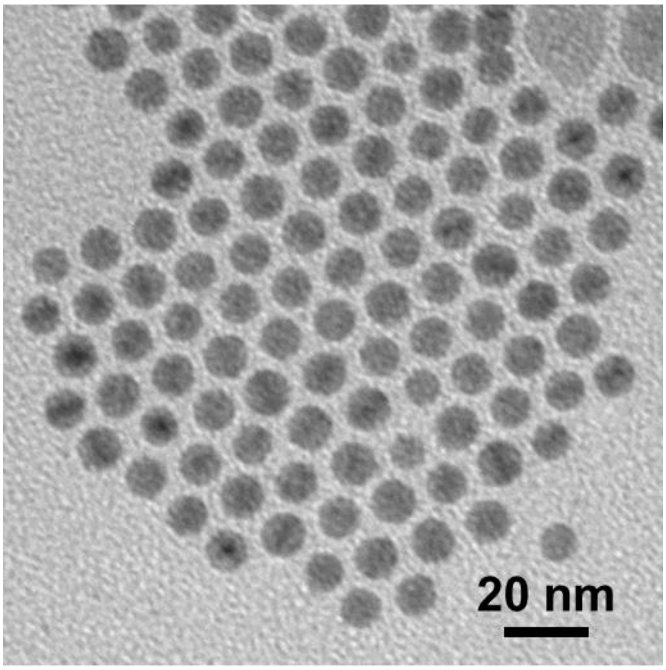

Supplementary Figure 27.
TEM image of the Ag NPs.

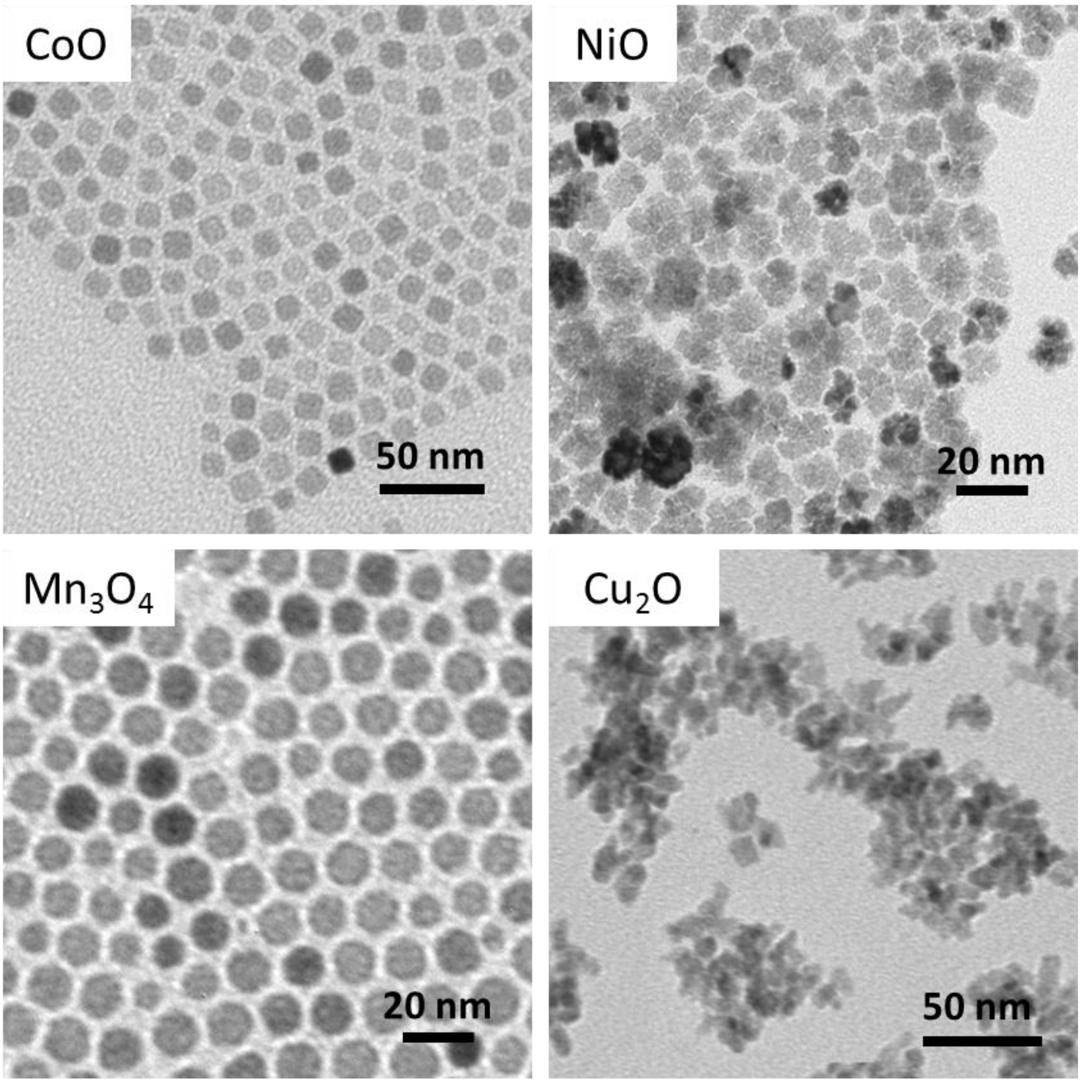

Supplementary Figure 28.

TEM images of as-synthesized transition metal oxide (TMO) NPs.

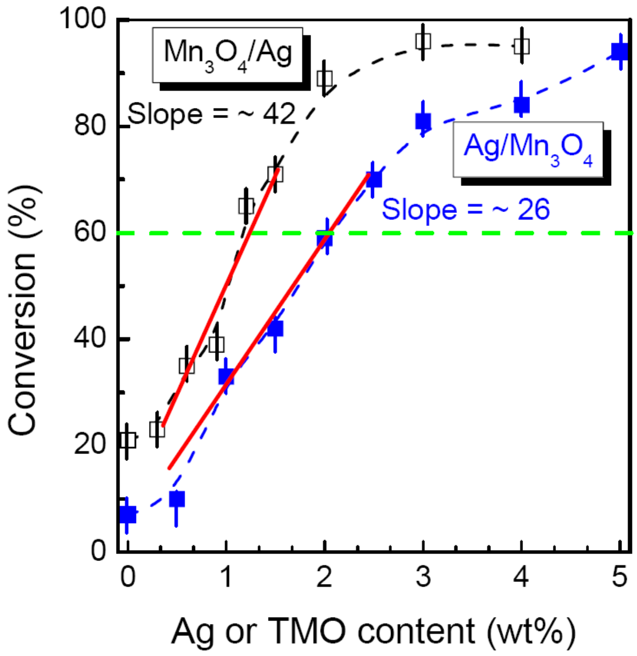

Supplementary Figure 29.

The catalytic performance of the catalyst nano-Ag/micro-TMO and nano-TMO/micro-Ag with different nano-Ag and nano-TMO contents. Reaction conditions: catalyst (0.15 g), Ag or Mn_3O_4 loading (4 wt%), WHSV (20 h^{-1}), air (50 mL/min), 270 ^oC.

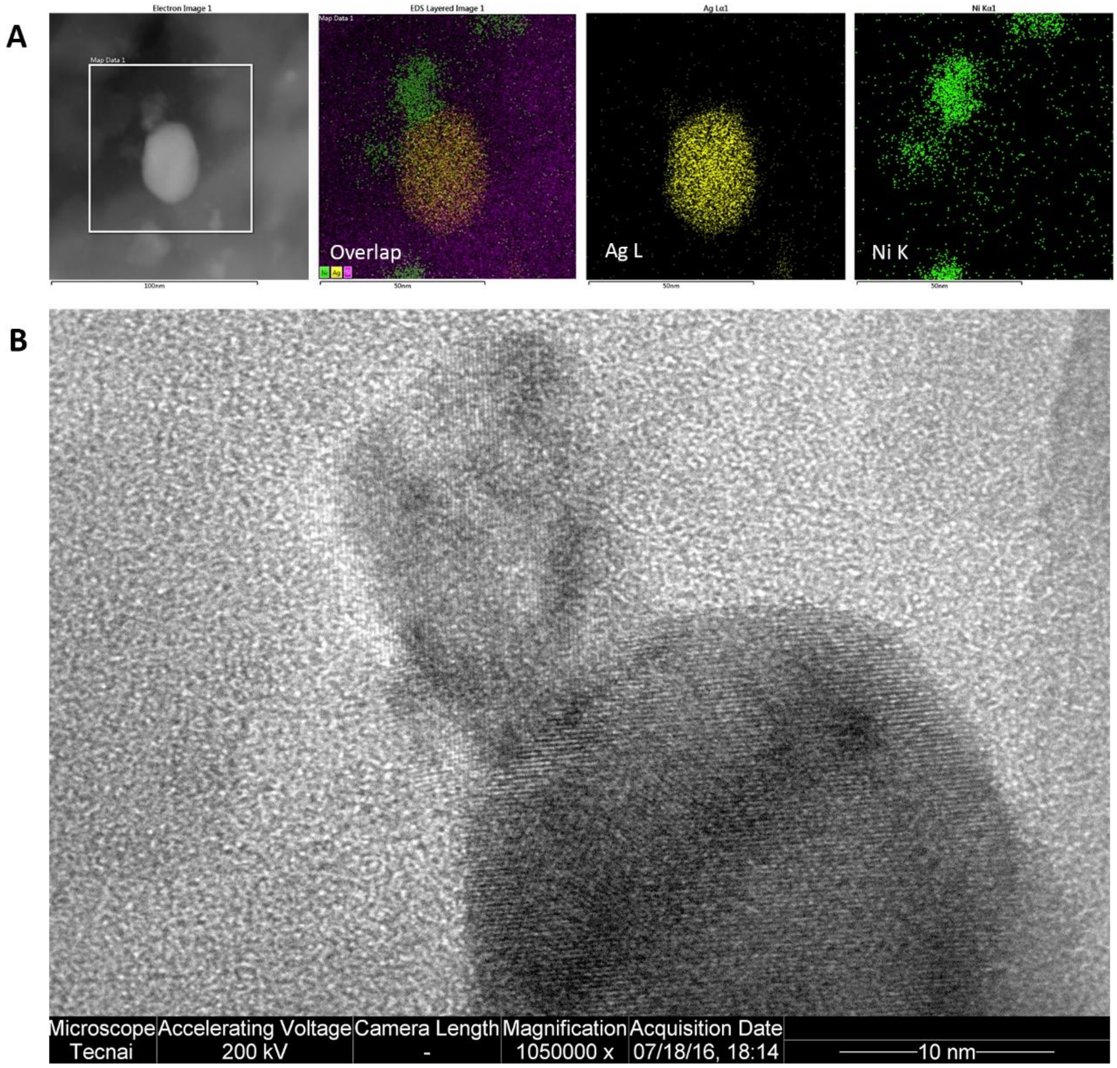

Supplementary Figure 30.

(A) HAADF-STEM and EDS elemental mapping images of the spent “Ag+NiO” physical mixtures. (B) HRTEM image of the sample in correspondence to the same particle in the EDS elemental mapping image.

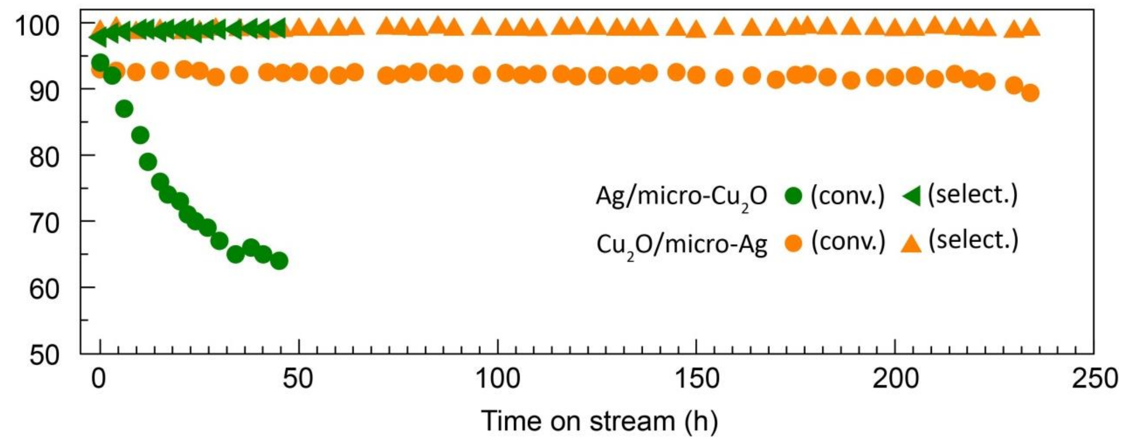

Supplementary Figure 31.

The stability test of nano-Cu_2O/micro-Ag and nano-Ag/micro-Cu_2O. Reaction conditions: catalyst (0.2 g), benzyl alcohol (4 g/h), air (60 mL/min), 0.1 MPa, 250 ^oC.

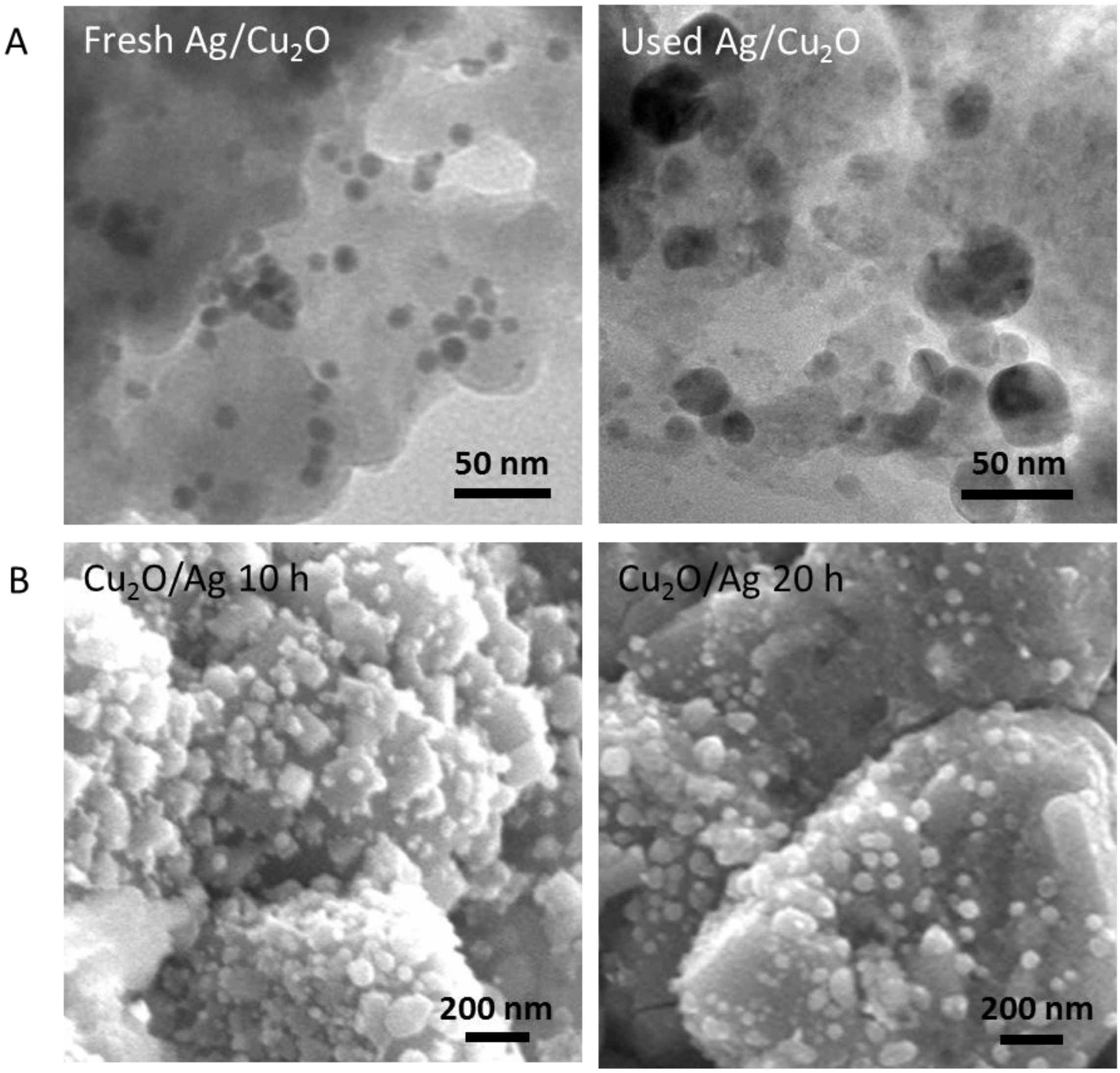

Supplementary Figure 32.

TEM images of the nano-Ag/micro-Cu_2O and nano-Cu_2O/micro-Ag catalysts. (**A-left**) Fresh nano-Ag/micro-Cu_2O and (**A-right**) Used nano-Ag/micro-Cu_2O after 30-h reaction. (**B-left**) Used nano-Cu_2O/micro-Ag after 10-h reaction and (**B-right**) Used nano-Cu_2O/micro-Ag after 10-h reaction. Reaction conditions: catalyst (0.15 g), Ag or Cu_2O loading (6 wt%), WHSV (20 h^{-1}), air (50 mL/min), 250 ^oC.

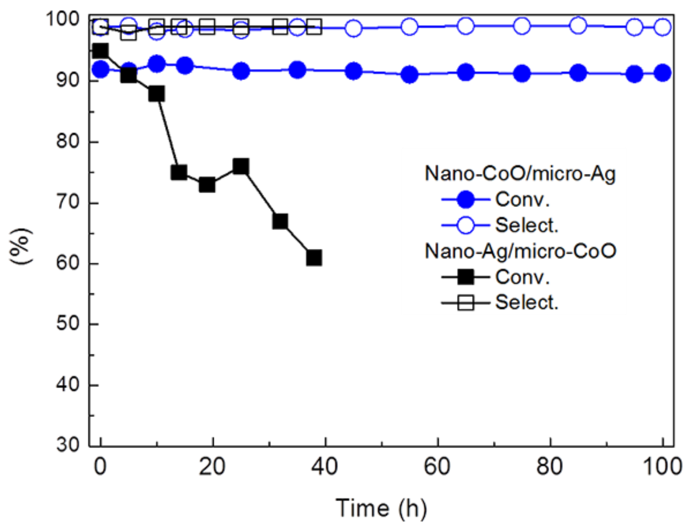

Supplementary Figure 33.

The stability test of nano-Ag/micro-CoO and nano-CoO/micro-Ag. Reaction conditions: catalyst (0.15 g), Ag or CoO loading (4 wt%), WHSV (20 h^{-1}), air (50 mL/min), 270 ^{o}C.

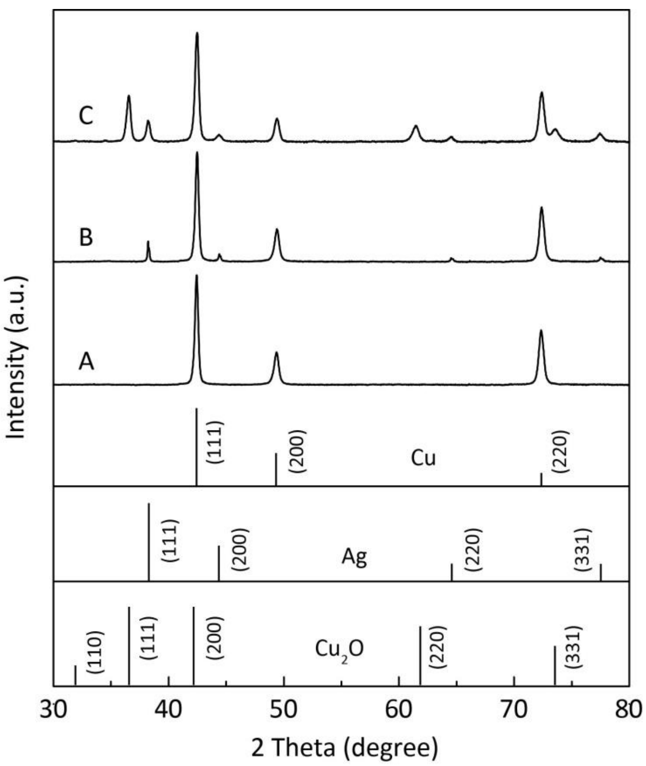

Supplementary Figure 34.

XRD patterns of the Cu-gauze (**A**), Ag/Cu-gauze (**B**) and Cu_2O/Ag/Cu-gauze (**C**).

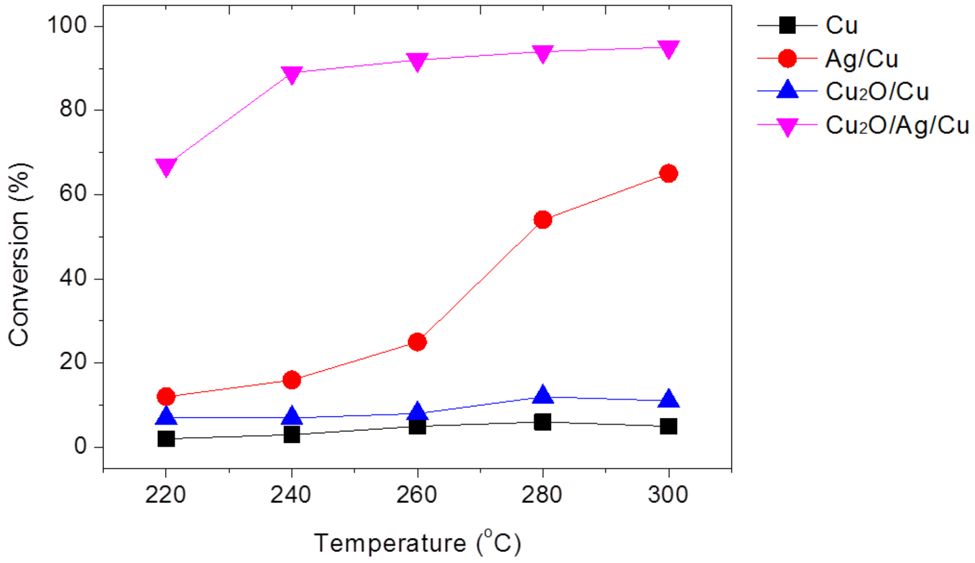

Supplementary Figure 35.

The catalytic performance of the Cu-gauze, Cu_2O/Cu-gauze, Ag/Cu-gauze and Cu_2O/Ag/Cu-gauze at different reaction temperatures. Reaction conditions: catalyst (1 g), WHSV (4 h^{-1}), air (60 mL/min).

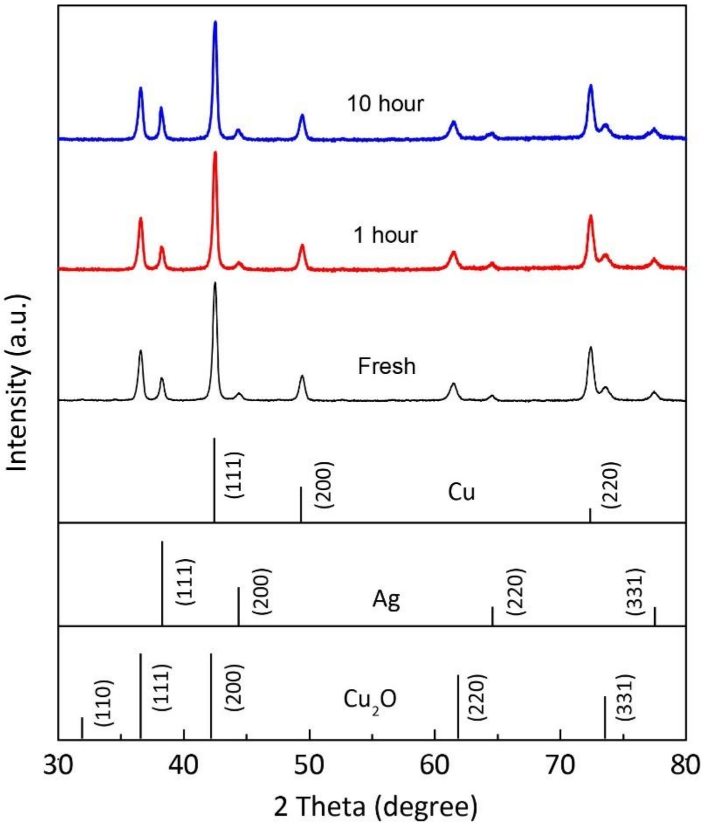

Supplementary Figure 36.

Evolution of XRD patterns of the Cu_2O/Ag/Cu-gauze during the benzyl alcohol oxidation..

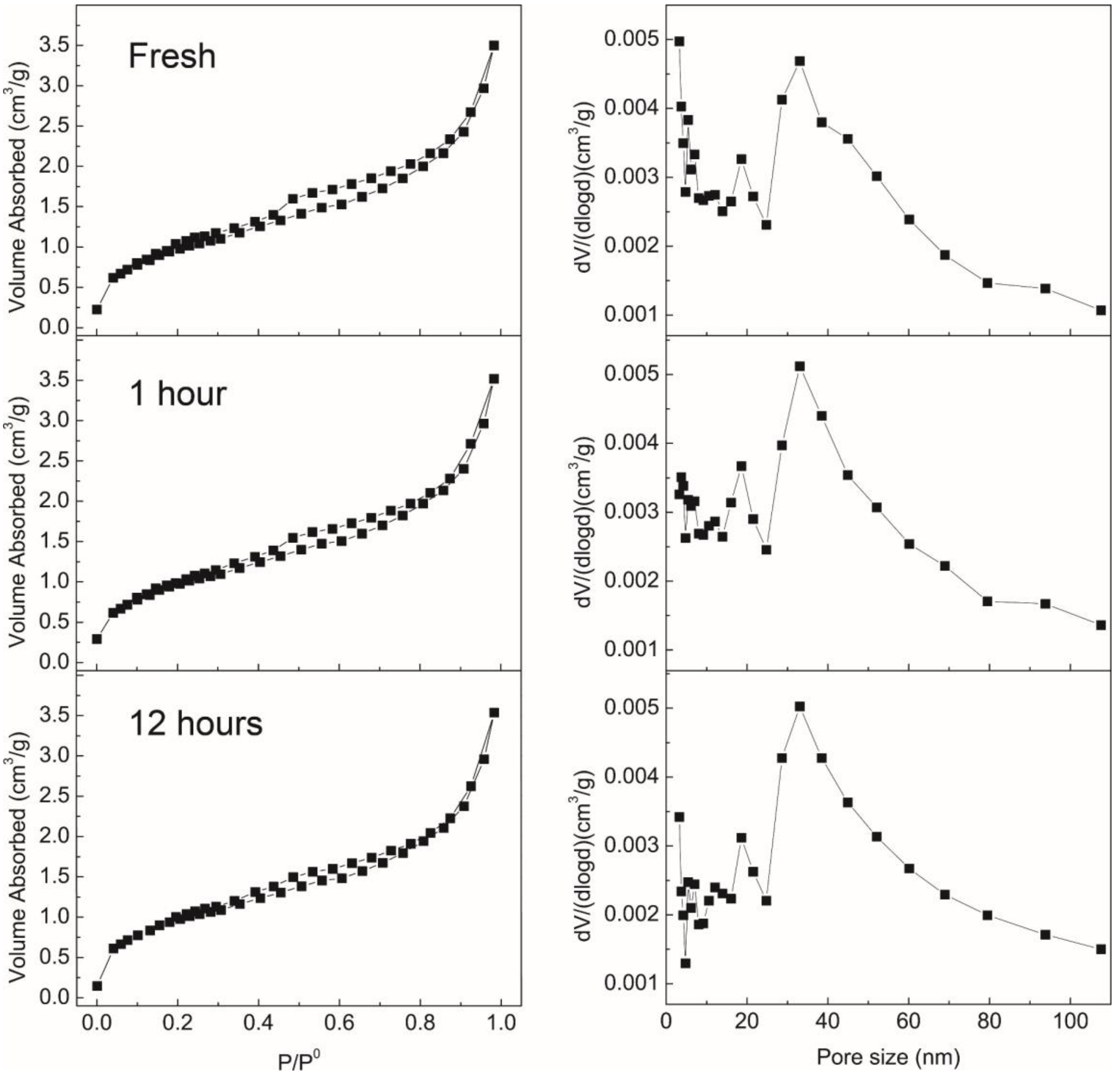

Supplementary Figure 37.

Evolution of the N_2 physical adsorption of the Cu_2O/Ag/Cu-gauze during the benzyl alcohol oxidation..

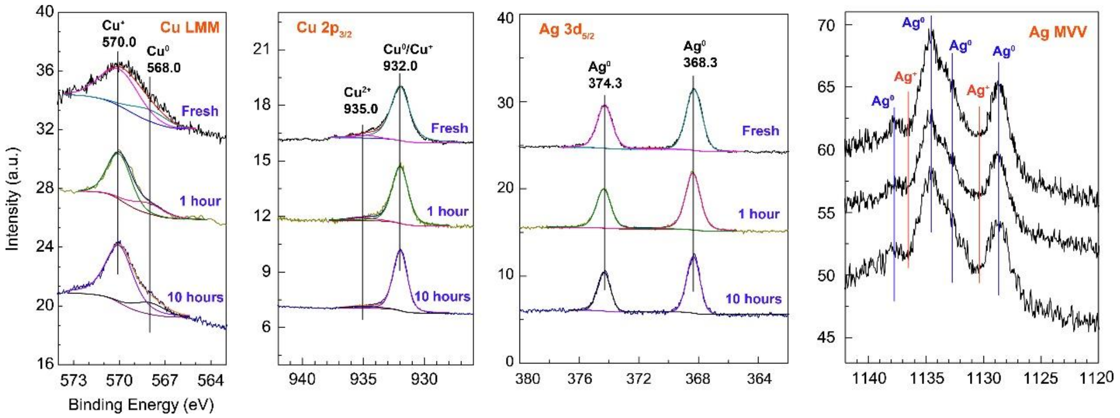

Supplementary Figure 38.

Evolution of the XPS of the Cu_2O/Ag/Cu-gauze during the benzyl alcohol oxidation..

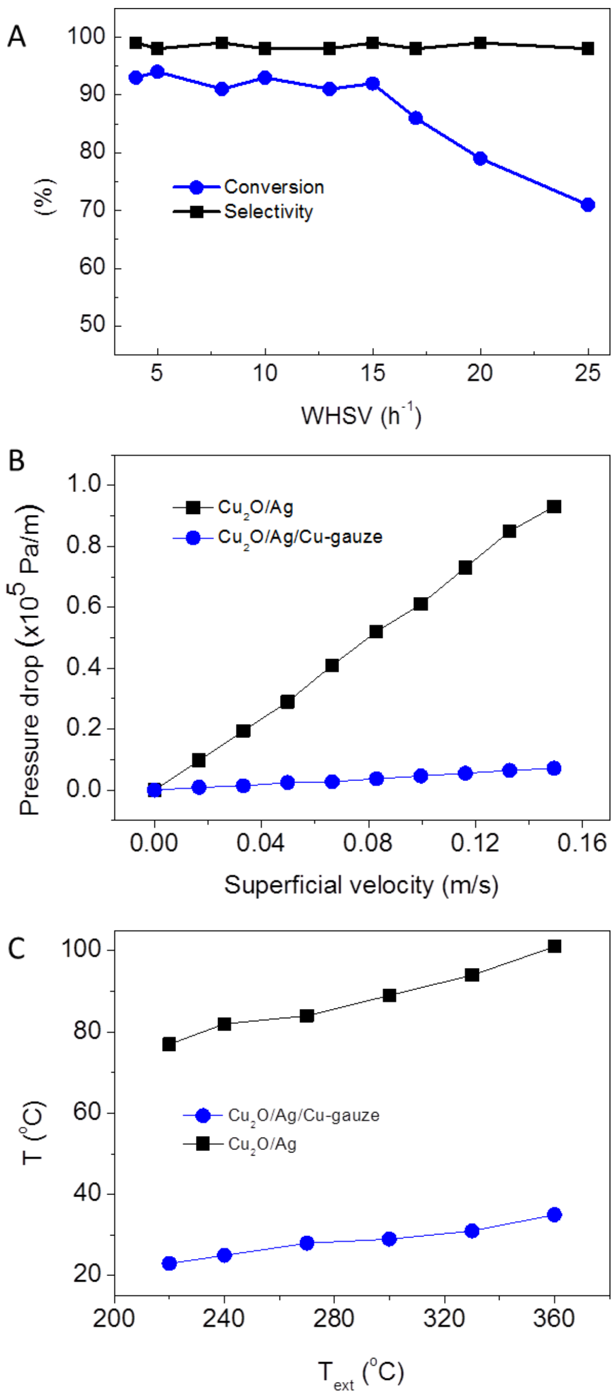

Supplementary Figure 39.

(**A**) The influence of WHSV on the catalyst performance of Cu_2O/Ag/Cu-gauze. Reaction conditions: catalyst (1 g), 300 °C, O_2/alcohol molar ratio (0.6), N_2 (50 mL/min). (**B**) Pressure drop of catalysts bed of Cu_2O/Ag/Cu-gauze and Cu_2O/Ag/SiO_2 (200 mesh). (**C**) Temperature difference (ΔT) between the setup temperature (T_{ext}) and the catalyst-bed temperature (T_{bed}) in the gas-phase oxidation of benzyl alcohol over the Cu_2O/Ag/Cu-gauze and Cu_2O/Ag/SiO_2.

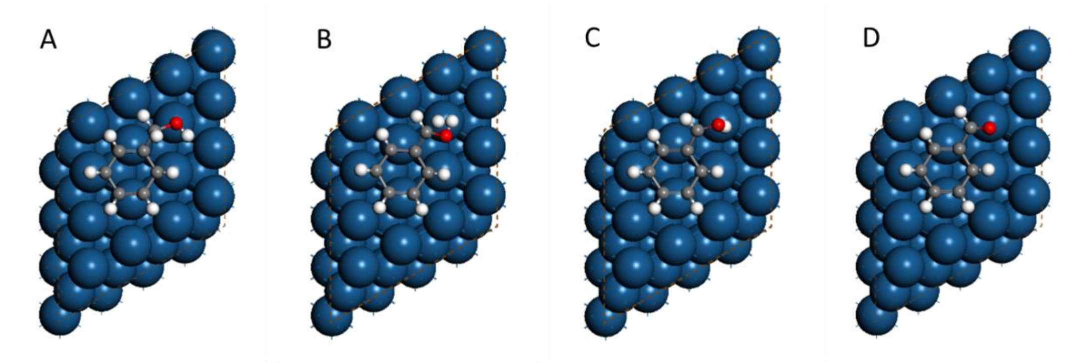

Supplementary Figure 40.

$C_6H_5CH_2OH$ oxidation beginning with C-H bond scission (pathway 2) on Pt(111). (**A**) Adsorption structures of $C_6H_5CH_2OH$. (**B**) Transition state structures of C-H bond activation (C-H distance: 1.60 Å). (**C**) Transition state structures of O-H bond activation (O-H and H-Pt distances: 1.28, 1.74 Å, respectively). (**D**) C_6H_5CHO adsorption. Blue balls represent Pt atoms, grey for C, white for H and red for O.

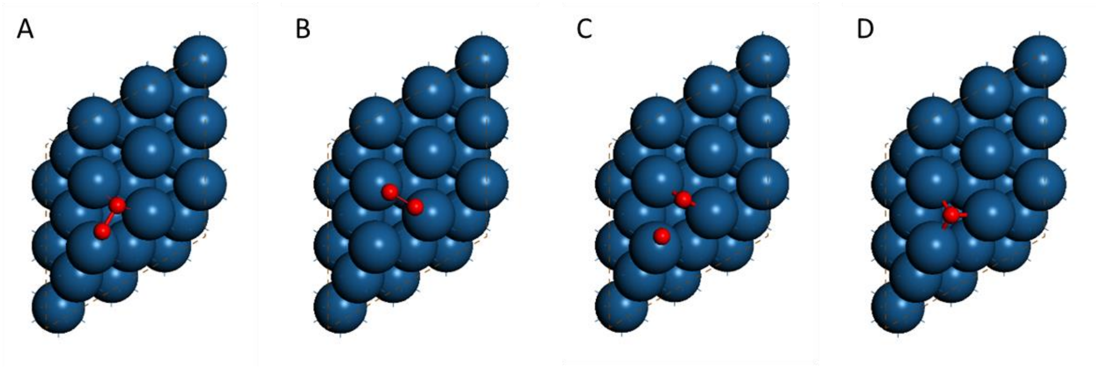

Supplementary Figure 41.

Adsorption and dissociation of molecular oxygen on Pt(111). (**A**) Adsorption of oxygen at bridge-top site (O-O distance: 1.40 Å, adsorption energy: -0.49 eV). (**B**) Adsorption of oxygen at top-top site (O-O distance: 1.35 Å, adsorption energy: -0.57 eV). (**C**) Transition state of oxygen dissociation (O-O distance: 2.00 Å, activation energy: 0.47 eV, relative to structure B). (**D**) Adsorption of O atom at *fcc* site (adsorption energy: -1.16 eV). Blue and red balls represent Pt and O atoms, respectively.

Supplementary Table 1.

Reaction and activation energies of $C_6H_5CH_2OH$ oxidation at the O-FeO/Pt(111) interface including the entropy contributions (1 bar, 270 ^oC).

Reactions		E_a / eV	ΔE / eV
Adsorption	$C_6H_5CH_2OH$ + * → $C_6H_5CH_2OH$ *	N/A	-1.06
Pathway 1	$C_6H_5CH_2OH$* + * → $C_6H_5CH_2O$* + H*	0.05	-0.39
	$C_6H_5CH_2O$* + * → C_6H_5CHO* + H*	0.14	-1.29
Pathway 2	$C_6H_5CH_2OH$* + * → C_6H_5CHOH* + H*	0.40	-1.60
	C_6H_5CHOH* + * → C_6H_5CHO* + H*	0.06	-0.07
Desorption	C_6H_5CHO* → C_6H_5CHO + *	N/A	0.65

Supplementary Table 2.

Oxygen vacancy formation with the aid of dissociated H atoms under experimental conditions (270 ^{o}C, 1 bar).

Reactions	E_a / eV	ΔE / eV
H* + OH* → H_2O (+ O_{v1})	0.05	-0.79
H* + OH* → H_2O (+ O_{v2})	0.39	-0.78

Supplementary Table 3.

Reaction energies and activation energies of $C_6H_5CH_2OH$ oxidation (pathway 1) on Pt(111) (-273 ^oC).

Reactions	Structure A in Supplementary Fig. 13		Structure E in Supplementary Fig. 13	
	E_a / eV	ΔE / eV	E_a / eV	ΔE / eV
$C_6H_5CH_2OH$ + * → $C_6H_5CH_2OH$*	N/A	-2.57	N/A	-0.80
$C_6H_5CH_2OH$* + * → $C_6H_5CH_2O$* + H*	0.70	0.29	0.77	0.64
$C_6H_5CH_2O$* + * → C_6H_5CHO* + H*	0.42	-0.30	N/A	N/A
C_6H_5CHO* → C_6H_5CHO + *	N/A	2.36	N/A	N/A

Supplementary Table 4.

Gas-phase oxidation of benzyl alcohol catalyzed by Pd and PdCu alloy catalysts[a].

Entry	Catalysts	Conversion (%)	Selectivity (%)[d]				
			A	B	C	D	E
1	Pd	20	87	9	2	1	1
2	Pd_3Cu	35	95	1.9	1.6	0.8	0.7
3	PdCu	46	96	1.4	1.3	0.7	0.6
4	$PdCu_3$	67	97	1.3	0.5	0.7	0.5
5	$PdCu_5$	93	97.5	0.6	0.5	0.9	0.5
6	$PdCu_7$	95	98	0.3	0.2	1.2	0.4
7	$PdCu_{10}$	94	98	0.2	0.2	1.3	0.3
8	Cu	21	99	0	0.1	0.6	0.3
9	Cu_2O[b]	19	99	0	0.1	0.7	0.2
10	Cu_2O+Pd[c]	91	95	2.1	1.5	0.9	0.5
11	Cu_2O+Pd_3Cu[c]	89	97.5	0.8	0.6	0.8	0.3
12	Cu_2O+PdCu[c]	93	98	0.4	0.2	0.2	0.2
13	Cu_2O+$PdCu_3$[c]	90	99	0.3	0.2	0.3	0.2

[a] Catalyst (0.2 g), total metal (Pd and Cu) loading (4 wt%) on SiO_2 (200 mesh), 270 ^{o}C, WHSV (weight hourly space velocity, 20 h^{-1}), air (60 mL/min). [b] Cu_2O loading (6 wt%). [c] Cu_2O (6 wt%) was physically mixed with Pd (4 wt%), Pd_3Cu (4 wt%), PdCu (4 wt%) or $PdCu_3$ (4 wt%) on silica support. [d] A: Benzaldehyde; B: Benzene; C: Toluene; D: Benzyl acid; E: CO_x.

Supplementary Table 5.

Activation energies for O-H and C-H cleavage of pathway 1 on M(111) surfaces (M = Pt, Pd, Ag).

	Pt(111)	Pd(111)	Ag(111)
$E_{a\ (O\text{-}H)}$ / eV	0.70	0.83	1.56
$E_{a\ (C\text{-}H)}$ / eV	0.42	0.38	0.56

Supplementary Table 6.

The catalytic performance of MgO/Ag at different temperatures.[a]

Entry	Temperature	Conv. (%)	Select. (%)
1	250	5.2	99
2	260	6.8	99
3	270	8.4	99
4	280	8.9	99
5	290	10.7	99
6	300	12.7	99

[a]Catalyst, 0.2 g; SiO_2 support, 200 mesh; air, 60 mL/min; Ag loading, 4-5 wt%; TMO loading, 3-4 wt%.

Supplementary Table 7.

Gas-phase oxidation of benzyl alcohol catalyzed by nano-Ag, nano-TMO, Ag-TMO, nano-Ag/micro-TMO, micro-Ag, and inverse nano-TMO/micro-Ag catalysts[a].

Entry	Catalyst	T (ºC)	Conv. (%)	Selec. (%)	TOF[i] (h^{-1})
1	$Ag\text{-}Cu_2O$[b-d]	230	94	99	8 155 ± 2 745[j]
2	Ag[b,e]	230	25	99	570 ± 74[k]
3	Cu_2O[b,f]	230	10	99	415 ± 35[l]
4	Ag+NiO[b-d]	230	91	99	7 370 ± 1 750[m]
5	$Ag+Mn_3O_4$[b-d]	230	95	98	7 775 ± 1 850[m]
6	Ag+CoO[b-d]	230	96	98	7 670 ± 1 830[m]
7	NiO[b,f]	230	9	99	430 ± 40[l]
8	CoO[b,f]	230	12	99	495 ± 35[l]
9	Mn_3O_4[b,f]	230	7	99	380 ± 30[l]
10	Ag/NiO[e,g]	250	92	98	7 220 ± 1 850[m]
11	Ag/CoO[e,g]	250	95	99	8 034 ± 2 060[m]
12	Ag/Cu_2O[e,g]	250	94	99	7 525 ± 1 930[m]
13	Ag/Mn_3O_4[e,g]	250	96	98	7 830 ± 2 008[m]
14	NiO/micro-Ag[f,h]	230	93	99	7 460 ± 2 390[n]
15	CoO/micro-Ag[f,h]	230	92	99	7 050 ± 2 050[n]
16	Cu_2O/micro-Ag[f,h]	230	91	99	6 440 ± 2 065[n]
17	Mn_3O_4/micro-Ag[f,h]	230	94	98	7 200 ± 1 970[n]
18	micro-Ag[h]	230	15	99	530 ± 120[o]
19	NiO[g]	250	3	98	387 ± 27[p]
20	CoO[g]	250	6	99	504 ± 36[p]
21	Cu_2O[g]	250	5	99	436 ± 51[p]
22	Mn_3O_4[g]	250	7	98	377 ± 33[p]

[a] WHSV (20 h^{-1}). [b] Catalyst (0.2 g), SiO_2 support (200 mesh), air (60 mL/min). [c] Ag loading (4-5 wt%). [d] TMO loading (3–4 wt%). [e] Ag loading (5 wt%). [f] TMO loading (4 wt%). [g] catalyst (0.15 g), air (50 mL/min). [h] micro-Ag (0.15 g), air (50 mL/min). [i] Turnover of frequency (TOF) of these catalysts was measured on the basis of benzyl

alcohol conversion of below 10% at 230 °C. [j] TOF of this catalyst was calculated on the basis of the amount of interfacial Ag sites with Cu_2O size of 23 ± 4 nm in the Ag-Cu_2O composites. [k] TOF of Ag NPs was calculated on the basis of the surface amount of Ag atoms with Ag size of 11.5 ± 1.5 nm. [l] TOF of these catalysts was calculated on the basis of the surface TMO units (surface area: NiO of 41-49 m^2/g, CoO of 37-43 m^2/g; Cu_2O of 40-48 m^2/g and Mn_3O_4 of 35-41 m^2/g). [m] TOF of these catalysts was calculated on the basis of the amount of interfacial Ag sites with Ag size of 11.5 ± 1.5 nm. [n] TOF of these catalysts was calculated on the basis of the amount of interfacial Ag sites with NiO size of 12.0 ± 2.0 nm, CoO size of 10.7 ± 1.7 nm, Cu_2O size of 12.0 ± 2.0 nm, and Mn_3O_4 size of 11.7 ± 1.7 nm. [o] TOF of this catalyst was calculated on the basis of the amount of surface Ag atoms (surface area of 1.3 ± 0.3 m^2/g). [p] TOF of these catalysts was calculated on the basis of the surface TMO units (surface area: NiO of 4.0-4.6 m^2/g, CoO of 3.9-4.5 m^2/g; Cu_2O of 3.0-3.8 m^2/g and Mn_3O_4 of 3.8-4.4 m^2/g).

Supplementary Table 8.

The activation energies of the catalysts[a].

Catalyst	Reaction order of O_2	Reaction order of benzyl	E_a (kJ/mol)
Cu_2O	0.06	0.52	87
Ag	0.07	0.67	68
Cu_2O-Ag	0.05	0.84	43

[a] The kinetic studies were conducted under the conditions that could eliminate the influence of heat/mass diffusion: catalyst (0.01 g), Ag or Cu_2O loading (4 wt%), WHSV (50 h^{-1}), O_2 (2 mL/min), N_2 (100 mL/min), SiO_2 support (200 mesh).

Supplementary Table 9.

Surface areas of different samples measured by N_2 physical adsorption at -196 ^oC.

Sample	Surface area (m^2/g)	Sample	Surface area (m^2/g)
Micro-Mn_2O_3	4.1	Nano-Ag-1.0 wt%/Micro-Mn_3O_4	35
Micro-Ag	1.1	Nano-Ag-1.5 wt%/Micro-Mn_3O_4	36
Nano-Mn_2O_3	37	Nano-Mn_3O_4-0.3 wt%/Micro-Ag	0.38
Nano-Ag	22	Nano-Mn_3O_4-0.6 wt%/Micro-Ag	0.51
Nano-Ag-0.5 wt% /Micro-Mn_3O_4	38	Nano-Mn_3O_4-0.9 wt%/Micro-Ag	0.6

Supplementary Table 10.

The catalytic performance of the spatially-separated physical mixtures of transition metal oxide and Ag.[a]

Entry	Catalyst	Conv. (%)	Select. (%)
1	Cu_2O/SiO_2 + Ag/SiO_2	31	99
2	NiO/SiO_2 + Ag/SiO_2	29	99
3	CoO/SiO_2 + Ag/SiO_2	34	99
4	Mn_3O_4/SiO_2 + Ag/SiO_2	28	99

[a]Catalyst, 0.2 g; SiO_2 support, 200 mesh; air, 60 mL/min; Ag loading, 4-5 wt%; TMO loading, 3-4 wt%; reaction temperature, 503 K; the Ag/SiO_2 and TMO/SiO_2 were physically mixed; the amounts of TMO and Ag in the TMO/SiO_2 and Ag/SiO_2 were kept the same as that of the Ag + TMO mixture catalyst (entry 4~6 in Supplementary Table 7).

Supplementary Table 11.

The amounts of metal precursors and the reaction temperatures for the synthesis of various nanoparticles

Catalyst	Metal precursor (mmol)	T (^{o}C)
Pt nanoparticles	$Pt(acac)_2$ / 0.1	270
Pd nanoparticles	$Pd(acac)_2$ / 0.1	190
PdCu nano-alloy	$Pd(acac)_2$ / 0.1 + $Cu(NO_3)_2{\cdot}6H_2O$ / 0.1	260
Ag-Cu_2O nanocomposites	$AgNO_3$ / 0.1 + $Cu(NO_3)_2{\cdot}6H_2O$ / 0.1	200
Ag NPs	$AgNO_3$ / 0.5	200
Cu_2O NPs	$Cu(NO_3)_2{\cdot}6H_2O$ / 0.5	220
NiO NPs	$Ni(NO_3)_2{\cdot}6H_2O$ / 0.34	250
CoO NPs	$Co(NO_3)_2{\cdot}6H_2O$ / 0.34	250
Mn_3O_4 NPs	0.1 g $Mn(NO_3)_2$ aqueous solution (50 wt%)	220

Supplementary Discussion

The free energy profiles of benzaldehyde formation on Pt(111)

Benzyl alcohol adsorbs mainly with the benzene ring sitting at the bridge(30) site (Supplementary Fig. 13A) (*1, 2*), which is strongly exothermic by 2.57 eV including the van der Waals (vdW) interactions between the adsorbate and the Pt surface (*3, 4*). The benzaldehyde formation can start with the O-H bond scission to remove the first hydrogen with an activation energy of 0.70 eV (pathway 1, Supplementary Fig. 13B). Subsequently, the second hydrogen can be removed by the C-H bond activation with a 0.42 eV barrier and 0.30 eV heat releasing. Alternatively, the C-H bond cleavage can firstly occur and then be followed by the O-H bond dissociation (pathway 2, Supplementary Fig. 40). In pathway 2, the activation energies of C-H and O-H cleavage (0.68 eV and 0.23 eV, respectively) are both lower than those in pathway 1, which implies that the pathway 2 would energetically be favorable for the benzaldehyde formation on Pt(111).

Pd and $PdCu_x$ NPs

Shown in Supplementary Fig. 14 are TEM images of the as-synthesized Pd NPs and $PdCu_x$ nano-alloy. The Pd NPs display worm-like morphology with diameters of 4–6 nm and lengths of 8–30 nm. The TEM images of spherical $PdCu_x$ nano-alloy with increased Cu/Pd mole ratio (e.g. 1/3, 1/1, 3/1, 5/1, and 7/1) were shown in Supplementary Fig. 14. The diffraction peaks in the XRD (Supplementary Fig. 17) of Pd NPs could be indexed to {111}, {200}, and {220} reflections (JCPDS No. 05-0681). With the increase Cu content in $PdCu_x$ alloy, the diffraction peaks shifted to higher 2θ degrees, in close to the characteristic diffractions of standard Cu (43.3^{o} {111}, 50.4^{o} {200}, and 74.1^{o} {200}, JCPDS No. 04-0836). XPS spectra of Pd and $PdCu_x$ NPs show that Pd and Cu existed dominantly in metallic state, with very little Pd^{2+}, Cu^{+} and Cu^{2+} (Supplementary Fig. 15).

Ag-Cu_2O nanocomposites

The XRD pattern of Ag-Cu_2O nanocomposite displays characteristic diffractions of Ag (JCPDS No. 4-0783) and Cu_2O (JCPDS No. 05-0667), demonstrating the sample comprises Ag and Cu_2O phases. High angle annular dark-field scanning transmission electron microscopy (HAADF-STEM) image (Supplementary Fig. 25) shows that the Ag phase (bright) and Cu_2O (dark) phase are close to each other in space. The corresponding EDX mappings (Supplementary Fig. 25) confirm the close contact of the two phase, indicating the formation of Ag-Cu_2O composite.

Supplementary Methods

Catalyst preparation

Pt and Pd nanoparticles (NPs), PdCu nano-alloy, Ag-Cu_2O nanocomposites, Ag, Cu_2O, NiO, CoO and Mn_3O_4 NPs were synthesized using procedures reported previously (*5*). In brief, 7.5 g of octadecylamine (ODA, Beijing Chemical Factory) was loaded in a 50 mL beaker and heated at 120 ^oC. Designed amounts of metal precursors were added in the ODA under magnetic stirring to form a clear solution. The reaction mixture was further heated to desired temperature (T) and maintained at this temperature for 10 min before it was cooled down to 70 ^oC. The resultant solution was mixed with 20 mL of ethanol and kept stand at 70 ^oC for 30 min without magnetic stirring. The products were collected at the bottom of the beaker by decanting the supernatant and further washed with hexane and ethanol for several times. The amounts of metal precursors and the reaction temperatures are listed in the Supplementary Table 11.

Cu NPs were prepared as described elsewhere (*6*). Briefly, 10 mL of trioctylamine (TOA) was loaded in a three-neck flask and heated at 130 °C for 30 min under a N_2 flow to remove dissolved water and oxygen. 1 mmol copper(I) acetate (CuOAc) and 0.5 mmol tetradecylphosphonic acid were added into TOA after cooling to room temperature. The solution was rapidly heated to 180 °C and maintained for 30 min under N_2 atmosphere. The solution was further rapidly heated to 270 °C, and held there for an additional 30 min before it was cooled to room temperature. 5 mL ethanol was added into the colloidal solution and the products were collected by centrifugation.

As-synthesized NPs were treated at 300°C in air to remove the organic capping agents.and loaded on the corresponding supports before they were subjected to catalytic investigation.

Details of density functional theory (DFT) calculations

The spin-polarised calculations were performed with the Perdew-Burke-Ernzerhof (PBE) functional within the generalised gradient approximation as implemented in the VASP package (*7, 8*). The project-augmented wave (PAW) method was used to represent the core-valence electron interaction (*9*). To model Pt(111), four-layer *p*(3×3) (Supplementary Fig. 41) and *p*(4×4) slabs (Supplementary Fig. 13) with 4 × 4 × 1 and 3 × 3 × 1 *k*-point samplings, respectively, were used. For the Fe-terminated FeO/Pt(111) surface, a FeO ribbon covered *p*(2√3 × 7) Pt(111) slab was used for benzyl alcohol oxidation (Supplementary Fig. 8B), which could further be oxidized to form the O-terminated FeO/Pt(111) surface. A 3 × 2 × 1 *k*-point sampling was applied and the on-site column repulsion was described by DFT + U with $U_{Fe} - J_{Fe}$ = 3 eV, as used in previous studies (*10*). A vacuum layer of 15 Å was applied and the bottom two layers were fixed for all the slabs. The valence electronic states were expanded in plane wave basis sets with a cutoff energy of 450 eV. Atomic positions were optimized until the maximum force of each atom was less than 0.05 eV/Å. The van der Waals (vdW) interactions between the adsorbate and the substrate surface were considered using the DFT-D3 method (*3, 4*). The benzene

adsorption was tested on $p(3\times3)$ Pt(111) and the result shows that the adsorption is exothermic by 2.06 eV by the D3 method, which was closed to the calculation value in the work of Tkatchenko (1.96 eV) by the PBE + vdW^{surf} (*1*) method and the experimental results in the same benzene coverage (1.57-1.91 eV) (*11*). The transition states were searched by using a constrained optimisation scheme (*12-14*) and were verified when (i) all forces on the atoms vanish and (ii) the total energy is a maximum along the reaction coordinate but a minimum with respect to the rest of the degrees of freedom. The vibration frequency calculations were performed for the transition state structures we obtained, which shows that the located TS structures are true saddle points. The adsorption energies $E_{ad(adsorbate)}$ were defined as $E_{ad(adsorbate)} = E_{adsorbate/surface} - E_{adsorbate} - E_{surface}$.

Supplementary References

1. W. Liu, J. Carrasco, B. Santra, A. Michaelides, M. Scheffler, A. Tkatchenko, Benzene adsorbed on metals: Concerted effect of covalency and van der Waals bonding. *Phys. Rev. B* **86**, 5050-5059 (2012).
2. M. Saeys, M.-F. Reyniers, G. B. Marin, M. Neurock, Density functional study of benzene adsorption on Pt(111). *J. Phys. Chem. B* **106**, 7489-7498 (2002).
3. S. Grimme, J. Antony, S. Ehrlich, H. Krieg, A consistent and accurate ab initio parametrization of density functional dispersion correction (DFT-D) for the 94 elements H-Pu. *J. Chem. Phys.* **132**, 154104-154119 (2010).
4. S. Grimme, S. Ehrlich, L. Goerigk, Effect of the damping function in dispersion corrected density functional theory. *J. Comp. Chem.* **32**, 1456-1465 (2011).
5. D. Wang, Y. Li, Effective octadecylamine system for nanocrystal synthesis. *Inorg. Chem.* **50**, 5196-5202 (2011).
6. L. Hung, C. Tsung, W. Huang, P. Yang, Room-temperature formation of hollow Cu_2O nanoparticles. *Adv. Mater.* **22**, 1910-1914 (2010).
7. G. Kresse, J. Furthmuller, Efficiency of ab-initio total energy calculations for metals and semiconductors using a plane-wave basis set. *Comp. Mater. Sci.* **6**, 15-50 (1996).
8. G. Kresse, J. Furthmuller, Efficient iterative schemes for *ab initio* total-energy calculations using a plane-wave basis set. *Phys. Rev. B* **54**, 11169-11186 (1996).
9. G. Kresse, D. Joubert, From ultrasoft pseudopotentials to the projector augmented-wave method. *Phys. Rev. B* **59**, 1758-1775 (1999).
10. S. Prada, L. Giordano, G. Pacchioni, C. Noguera, J. Goniakowski, Properties of Pt-supported iron oxide ultra-thin films: Similarity of Hubbard-corrected and hybrid density functional theory description. *J. Chem. Phys.* **141**, 144702-144702 (2014).

11. H. Ihm, H. M. Ajo, J. M. Gottfried, P. Bera, C. T. Campbell, Calorimetric Measurement of the Heat of Adsorption of Benzene on Pt(111). *J. Phys. Chem. B* **108**, 14627-14633 (2004).

12. A. Alavi, P. Hu, T. Deutsch, P. L. Silvestrelli, J. Hutter, CO oxidation on Pt(111): An ab initio density functional theory study. *Phys. Rev. Lett.* **80**, 3650-3653 (1998).

13. A. Michaelides, P. Hu, Catalytic water formation on platinum: A first-principles study. *J. Am. Chem. Soc.* **123**, 4235-4242 (2001).

14. Z. Liu, P. Hu, General rules for predicting where a catalytic reaction should occur on metal surfaces: A density functional theory study of C-H and C-O bond breaking/making on flat, stepped, and kinked metal surfaces. *J. Am. Chem. Soc.* **125**, 1958-1967 (2003).
